# Common and mutation specific phenotypes of *KRAS* and *BRAF* mutations in colorectal cancer cells revealed by integrative -omics analysis

**DOI:** 10.1186/s13046-021-02025-2

**Published:** 2021-07-07

**Authors:** Snehangshu Kundu, Muhammad Akhtar Ali, Niklas Handin, Louis P. Conway, Veronica Rendo, Per Artursson, Liqun He, Daniel Globisch, Tobias Sjöblom

**Affiliations:** 1 Science For Life Laboratory, Department of Immunology, Genetics and Pathology, Uppsala University, SE-751 85 Uppsala, Sweden; 2 Present affiliation: School of Biological Sciences, University of the Punjab, Lahore, Pakistan; 3 Department of Pharmacy, Uppsala University, SE-751 23 Uppsala, Sweden; 4 Department of Chemistry - BMC, Uppsala University, SE-751 24 Uppsala, Sweden

**Keywords:** Ras pathway, *KRAS*, *BRAF*, Colorectal cancer, Isogenic cell models, Integrative -omics analysis

## Abstract

**Background:**

Genes in the Ras pathway have somatic mutations in at least 60 % of colorectal cancers. Despite activating the same pathway, the *BRAF* V600E mutation and the prevalent mutations in codon 12 and 13 of *KRAS* have all been linked to different clinical outcomes, but the molecular mechanisms behind these differences largely remain to be clarified.

**Methods:**

To characterize the similarities and differences between common activating *KRAS* mutations and between *KRAS* and *BRAF* mutations, we used genome editing to engineer *KRAS* G12C/D/V and G13D mutations in colorectal cancer cells that had their mutant *BRAF* V600E allele removed and subjected them to transcriptome sequencing, global proteomics and metabolomics analyses.

**Results:**

By intersecting differentially expressed genes, proteins and metabolites, we uncovered (i) two-fold more regulated genes and proteins when comparing *KRAS* to *BRAF* mutant cells to those lacking Ras pathway mutation, (ii) five differentially expressed proteins in *KRAS* mutants compared to cells lacking Ras pathway mutation (IFI16, S100A10, CD44, GLRX and AHNAK2) and 6 (CRABP2, FLNA, NXN, LCP1, S100A10 and S100A2) compared to *BRAF* mutant cells, (iii) 19 proteins expressed differentially in a *KRAS* mutation specific manner versus *BRAF* V600E cells, (iv) regulation of the Integrin Linked Kinase pathway by *KRAS* but not *BRAF* mutation, (v) regulation of amino acid metabolism, particularly of the tyrosine, histidine, arginine and proline pathways, the urea cycle and purine metabolism by Ras pathway mutations, (vi) increased free carnitine in *KRAS* and *BRAF* mutant RKO cells.

**Conclusions:**

This comprehensive integrative -omics analysis confirms known and adds novel genes, proteins and metabolic pathways regulated by mutant *KRAS* and *BRAF* signaling in colorectal cancer. The results from the new model systems presented here can inform future development of diagnostic and therapeutic approaches targeting tumors with *KRAS* and *BRAF* mutations.

**Supplementary Information:**

The online version contains supplementary material available at 10.1186/s13046-021-02025-2.

## Background

Activating mutations in the Ras pathway contribute to tumorigenesis in a wide array of human cancers. In colorectal cancers (CRC), somatic mutations in *KRAS*, *BRAF*, or *ERBB* receptors occur in ~ 60 % of patient tumors. The proto-oncogene *KRAS* encodes a member of the p21 Ras family of monomeric GTPases. Under physiological conditions, KRAS is activated through GDP:GTP exchange upon binding of growth factors such as EGF to their cognate tyrosine kinase receptors, which then allows KRAS to bind and activate downstream effectors such as BRAF. Somatic *KRAS* and *BRAF V600E* mutations are mutually exclusive in CRC [1], i.e. no further selective advantage is provided by the second mutation when the first mutation is already present. Whereas V600E is the predominant mutation in *BRAF*, several amino acids are frequently found altered in *KRAS*. Of these, codon 12 and 13 mutations are found in ~ 80 % of all CRC cases with *KRAS* mutation (G12D > G12V > G13D > G12C) [2]. It has been proposed that disease aggressiveness as well as response to therapy depends on the type of amino acid substitution in KRAS, where CRCs with *KRAS* G12D mutations had worse prognosis and were less responsive to anti-EGFR therapy as compared to those with G13D mutations [3]. Similarly, the phenotype of the *BRAF V600E* mutation was different from those of *KRAS* mutations. Activating mutations in *KRAS* support the decoupling of glycolysis and TCA metabolism, with glutamine supplying increased carbon to drive the TCA cycle [4]. Several studies have compared the effects of *KRAS* and *BRAF* mutations in genome edited cell systems [5, 6]. An analysis of *KRAS G12D*, *G12V* and *G13D* in human SW48 CRC cells revealed differential reprogramming of proteomes between KRAS codon 12 and 13 mutant cell lines. Specifically, DCLK1, AKAP12, c-MET and Caveolin-1 were upregulated in *KRAS* codon 12 mutant cell whereas ZO-2 (tight-junction protein) and ALDH3A1 were upregulated in *KRAS* codon 13 mutant cells [7]. Studies of isogenic non-small-cell lung cancer cell lines harboring different *KRAS G12C, G12D*, or *G12V* mutations revealed differential metabolic signatures related to enhanced amino acid catabolism, altered lipid biochemistry and altered antioxidant program [8]. CRC cell lines with *BRAF V600E* but not those with *KRAS G12C*, *G12D*, *G12V*, or *G13D* mutation responded to siRNA mediated BRAF inhibition by reduced proliferation and increased apoptosis [9], indicating that there are other branches of KRAS signaling that can support cell survival and proliferation. However, the underlying differences in molecular mechanisms between mutant *KRAS* and *BRAF* signaling are still largely unknown. Prior studies have either (i) been performed in cells originally lacking mutations in *KRAS* or *BRAF*, such as SW48, thus being independent of these mutations for tumor formation [3], (ii) compared *KRAS* and *BRAF* mutations in different genetic backgrounds [5, 9], or (iii) been limited to characterizing either the transcriptome, proteome or metabolome without integrative approach. To directly compare the effects of *KRAS* and *BRAF* mutations in a genetic background originally dependent on pathway mutation, we engineered *KRAS G12C*, *G12D*, *G12V* and *G13D* mutations in colorectal cancer RKO cells where the mutant *BRAF V600E* allele had been removed. As significant alterations at the transcriptome, proteome or metabolome level could lead to better understanding of the Ras pathway, useful cancer biomarkers, or guide drug discovery efforts, the engineered cell systems were characterized by transcriptome sequencing, global proteomics and metabolomics.

## Methods

### Knock-in of Ras pathway mutations by genome editing

Genome editing was performed using recombinant AAV-mediated homologous recombination. Both the *KRAS* and *BRAF* mutations were included in the homology arm one (HA1) for each corresponding gene targeting construct. The HA1s were PCR amplified directly from the genomic DNA of cell line where they were intended to be used later, to avoid including SNP variations. The mutations were engineered in the HA1s by using primers carrying these mutations in overlapping PCR (stitching PCR) used to amplify the mutant HA1s. Using the Gateway cloning system, Homology Arms (HAs) and selection cassette were cloned into a destination vector to make the gene targeting construct [10]. The primers used for construction and integration screening are listed in Supplementary Tables 1, Additional File 2. Phusion DNA polymerase (Finnzyme) was used to amplify HAs using *attB* tailed primers 1–4. The PCR conditions were initial denaturation at 98 °C for 3 min, 3 cycles of denaturation at 98 °C for 20 s, annealing at 64 °C for 20 s and extension at 72 °C for 30 s, followed by three cycles at 61 and 58 °C annealing temperature, respectively. The final amplification had 25 cycles of denaturation at 98 °C for 20 s, annealing at 57 °C for 20 s and extension at 72 °C for 30 s. Next, 100 ng each of HA1 and HA2 PCR products were recombined with 150 ng of pDONR™ P1-P2 and pDONR™ P3-P4 respectively, using BP Clonase II (Invitrogen, 11789-020) according to the manufacturer’s instructions. The resulting entry clones were screened for the presence of HAs by colony PCR amplification using Platinum *Taq* DNA polymerase (Invitrogen) and M13 primers 5–6 flanking the cloned HAs in the pDONR vectors. The PCR products from the colony PCR for pEntry-HA1 were sequenced to ensure the presence of *KRAS G13D* mutation in HA1. Next, 10 fmol of each of pEntry-HA1, pBUOY.SV40.HA.GFP.Myc.BsdpA [10], and pEntry-HA2 vectors were recombined with 15 fmol of pAAV-Dest vector using LR Clonase II (Invitrogen) according to the manufacturer’s instructions. The correct orientations of all the three components in the final targeting construct were confirmed by colony PCR using LR screening primers 7–10. AAV virus particles were generated as described [10]. RKO BRAF WT cells [5] (Horizon Discovery Ltd) were maintained in McCoy’s 5 A medium (Life Technologies) supplemented with 10 % fetal bovine serum and 1 % penicillin-streptomycin (Invitrogen) at 37 °C and 5 % CO_2_. Six million cells were seeded in a 75 cm^2^ flask and lysate containing the rAAV. Virus particles for KRAS G13D, G12C, G12V, or G12D were applied directly to the cells in 4 ml of complete growth medium. Forty-eight hours after infection, the cells were harvested and seeded at limiting dilution into twenty 96-well plates in selection medium containing G418 at 450 µg/ml for HCT116 cells and 800 µg/ml for RKO cells. The cells were grown under selection for 4 weeks and the resulting clones were screened for site specific integration of the targeting construct. The clones were harvested in 25 µl of trypsin without phenol red, 5 µl of which were lysed in 12 µl of Lyse-N-Go reagent (Thermo Scientific) and screened for site specific integration by using PCR with Platinum *Taq* DNA polymerase, 1 µl of Lyse-N-Go lysate and primers 11–12. The PCR products from positive cell clones were sequenced to confirm mutation engineering using primer 12. To excise the selection cassette from the targeted allele in positive clones, 0.5 million cells were seeded in 25 cm^2^ flasks and the growth medium was replaced by 4 ml of medium containing 10^7^ plaque forming units of Ad-CMV-Cre-GFP (Vector Biolabs). The cells were incubated for 24 h and were then seeded into 96-well plates at limiting dilution to get single cell clones. The clones were grown for 4 weeks and then screened for the removal of selection cassette using Lyse-N-Go sample preparation and primers 13–14. The PCR products were sequenced to confirm all the *KRAS* mutations and *BRAF V600E* in the respective targeted alleles using primers 15–18.

### Validation of Ras pathway mutation status in knock-in cell clones

To confirm the introduction of *KRAS* and *BRAF* mutations, RNA was extracted from RKO and HCT116 cells using the RNeasy Plus Mini Kit (cat. No. 74,136, Qiagen). Remaining genomic DNA was removed by incubation with DNase I (cat. No. AM2222, Thermo Scientific) for 30 min at 37 °C, followed by 10 min at 75 °C. Next, cDNA was synthesized from 2 µg template RNA using the Maxima H Minus First Strand cDNA Synthesis Kit (cat. No. K1652, Thermo Scientific), using random hexamer primers. Primers covering the KRAS and BRAF regions of interest were designed for an amplified product size of 225 and 266bps (Supplementary Tables 1, Additional File 2), respectively. Amplification of the *KRAS/BRAF* regions of interest was performed in 20 µL PCR reactions containing 1 × Phusion HF Buffer (Thermo Scientific), 0.2 mM dNTPs, 0.2 µM forward and 0.2 µM reverse primers, 0.02 U/µL Phusion DNA Polymerase (Thermo Scientific) and 6 ng of cDNA. The PCR was performed in a 2720 Thermal Cycler (Applied Biosystems) and consisted of 1 cycle of 98 °C for 30 s and 30 cycles of 98 °C for 15 s, 60 °C for 20 s and 72 °C for 30 s followed by 1 cycle of 72 °C for 10 min. For sequencing the obtained PCR products, 17 µL reactions were prepared containing 15 ng of the template DNA and the corresponding forward or reverse amplification primer (20 pmol). Sanger sequencing was performed at Eurofins Genomics. The obtained sequences were analyzed for mutations of interests using SnapGene Viewer 4.2.3.

*RNA sequencing*. The parental and isogenic cell lines were plated and grown to 70–80 % confluency in one T75 flask each. The cells were washed thrice with ice cold PBS and detached with cell scrapers. Approximately one third of the total cells were pelleted for RNA isolation. Total RNA was prepared using the Qiagen RNeasy Mini kit with on-column DNase treatment. RNA quality and concentration was determined by analysis on a Bioanalyzer 2100 instrument using the RNA 6000 nanochip (Agilent) and the samples were sequenced by paired-end sequencing on an Illumina HiSeq2500 sequencer. The analysis was based on raw sequencing data in fastq format. Quality and adaptor trimming was carried out using TrimGalore version 0.6.4 [11] in paired-end mode using Cutadapt version 2.4 [12]. A Phred score cut off of 20 was used and all reads over 20 bp long containing less than 10 % errors were retained. Only matched pairs were retained. Quality of the RNA-seq reads and the trimmed reads was inspected using FastQC software version v0.11.8 [13]. RNA-seq reads were aligned to the hsa genome (hg38) and annotated based on the corresponding annotation reference file, using STAR aligner, version 2.7.3 [14]. Gene-level read counts were obtained simultaneously with the alignment process using the quant mode of STAR. The read counts were normalized using EBseq R package [15]. Following normalization, Differential Expression Analysis was performed between knock-ins of *KRAS* mutations as compared to isogenic controls using the same package. Genes that resulted in an absolute log_2_ fold change > 1, and a False Discovery Rate (FDR) < 0.05 were considered differentially expressed. Principal Component Analysis of the normalized data was performed using the prcomp function from base R, and visualised using the ggfortify R package [16]. Normalized expression of the differentially expressed genes of each comparisons were visualized in heatmaps using the pheatmap R package [17]. Both samples and genes were clustered by Euclidian distances, and row-wise scaling was applied to the visualizations. Venn analysis and visualizations were performed using the VennDiagram R package [18].

### Identification of differentially expressed genes in the TCGA RNAseq dataset from colon and rectum

The expression data from TCGA colon (COAD dataset, 17 *KRAS* G12, 4 *KRAS* G13 and 28 non-*KRAS* mutant tumors) and rectal cohorts (READ dataset, 6 *KRAS* G12, 2 *KRAS* G13, 1 *KRAS* G61 and 8 non-*KRAS* mutant tumors), accessed on May 13, 2020, was divided into two groups, those that had *KRAS* 12 C/D/V, G13D and *BRAF* V600E mutations or those lacking such mutations, followed by analysis using EbSEq. All samples not tested for KRAS mutation were excluded. The resulting DEGs were compared to those identified in the RKO cell models.

### Proteomic analyses by liquid chromatography-tandem mass spectrometry

Cell pellets with 10^6^ cells were lysed in 0.1 M HEPES (pH 7.8), 2 % sodium dodecyl sulfate, and 0.05 M dithiothreitol for 5 min at 100 °C. The lysates were sonicated with a Branson-rod-type and then centrifuged at 14,000×*g* for 10 min to clarify the lysates. Samples were then processed in 30-kDa ultrafiltration units with the MED-FASP [18] protocol with the modification of substituting TRIS with HEPES. Lys-C and trypsin was used for the digestion. The digests were concentrated using a GeneVac EZ-2plus at 37 °C and peptide concentration were measured with the tryptophan fluorescence assay [19]. Tandem mass tag (TMT) labelling was performed according to the manufacturer’s instructions. The peptide samples were analysed in technical duplicate on an UltiMate 3000 RSLCnano system coupled to a QExactive HF mass spectrometer (Thermo-Fisher Scientific, Palo Alto, CA). An EASY-Spray 50-cm C18 column with a 75-µm inner diameter at 55 °C was used with a 240 and 200 min H_2_O-Acetonitrile gradient (0.1 % formic acid). The QExactive HF was set to data-dependent mode (Full MS/dd-MS^2^) with the survey scan and MS^2^ scan set at a resolution of 120,000 and 45,000, respectively. The top 15 most abundant peaks with a charge of 2–7 from the survey scan (375 to 1800) were selected with an isolation window of 1.0 m/z and fragmented by normalized collision energy of 34.5. The maximum ion injection times for the survey and MS2 scan were 20 and 100ms, respectively. The dynamic exclusion was set to 90 s and the acquired MS data were analysed using MaxQuant software (version 1.6.3.3). Proteins were identified by searching MS and MS/MS data of peptides with a fragment ion mass tolerance of 10ppm and parent ion tolerance of 2.5ppm against the human proteome reference database from UniProt (October 2018). The protein and peptide false discovery rates (FDRs) were set to 1 %. The data were first filtered, and entries with either the “Reverse”, “Potential.contaminant” or the “Only.identified.by.site” tags were removed before further processing. The limma R package [19] was used to remove the batch effect discovered after performing Principal Component Analysis. The data was normalized using quantile normalization and log2 transformation was applied. The limma R package was used to perform the differential expression analysis. *P*-values were adjusted using Benjamini-Hochberg procedure [20]. Proteins that displayed an absolute log2 fold change higher than 1 and an adjusted p-value lower than 0.05 were considered to be differentially expressed. For the visualizations of the Proteomics data, Principal Component Analysis (PCA) of the normalised data was performed using the prcomp function from base R, and visualised using the ggfortify R package [16]. Normalized expression of the differentially expressed genes of different comparisons between knock-ins of *KRAS* mutations as compared to isogenic controls were visualized as heatmaps using the pheatmap R package [17]. Both samples and genes where clustered by euclidian distance, and row-wise scaling was applied. Venn analysis and visualizations were performed using the VennDiagram R package [18].

### IPA canonical pathway analysis of transcriptomic and proteomic data

Ingenuity Pathway Analysis (IPA) [21] was used for pathway enrichment analysis of proteomics dataset. The full sets of measured proteins were used as reference sets in all analyses. Differentially expressed proteins (DEPs) were defined using the following cutoff criteria: adjusted *p*-value < 0.05 and absolute log2 fold change > 1. The significance values (*p-*value of overlap) for the IPA Canonical Pathways were calculated by the right-tailed Fisher’s Exact Test, and the p-values were adjusted for multiple testing using the Benjamini-Hochberg procedure [20]. The ratio of the number of DE molecules associated with a given pathway divided by the total number of molecules in the reference set that map to the pathway was calculated. IPA also calculated for each pathway a z-score that predicted pathway activation if positive or inhibition if negative. The z-score is calculated by comparing the dataset fold changes under analysis with the canonical pathway patterns in the IPA Knowledge Base. Z-scores of ≥ 2 or ≤ -2 were considered significant, and no z-score annotation indicates either zero (or very close to zero) z-score or that the given pathway is ineligible for a prediction. The IPA Overlay tool was used for overlaying the expression fold change values of the DE analysis. The IPA Molecule Activity Prediction (MAP) tool was then used for predicting down-stream and up-stream activation or inhibition of molecules and/or processes based on the observed expression changes. Additionally, heatmaps (using R package heatmap and Binary values representing enriched or not) that could compare the IPA results for the different mutations were created using the significantly enriched Pathway results for transcriptome datasets for all the knock-ins of different mutants as compared to their corresponding isogenic controls (Table 1) as well as for the proteomics datasets for the G12D and G13D as only these samples had enough differentially expressed proteins to be processed by IPA.

**Table 1:** Knock-in cell models and isogenic control cells. Knock-in cell models were generated with rAVV mediated gene editing. Mutation status for KRAS and BRAF oncogene genes in each of the isogenic knock-ins and control cell lines were validated by Sanger sequencing

Designations	Clone	BRAF c.1799T> A (V600E)	BRAF genotype	KRAS				KRAS genotype	Reference
				c.38G>A (G13D)	c.34G>T (G12C)	c.35G>T (G12V)	c.35G>A (G12D)		
HCT116	Parental	T	wt	G/A	G	G	G	wt/G13D	(Brattain et al. 1981 [22])
HCT116 KRAS WT	DK(HD)	T	Wt	G	G	G	G	wt/KO	(Shirasawa et al. 1993) [24]
HCT116 BRAF V600E	MHB3.2	T/A	wt/V600E	G	G	G	G	wt/KO	This work
RKO	Parental	T/A/A	wt/V600E/ V600E	G	G	G	G	wt/wt	(Brattain et al. 1984 [23])
RKO BRAF WT	RB(HD)	T	wt/KO/KO	G	G	G	G	wt/wt	(Yun et al. 2009) [5]
RKO KRAS G13D	MH2.1	T	wt/KO/KO	G/A	G	G	G	wt/G13D	This work
	MH3.1	T		G/A	G	G	G		
RKO KRAS G12C	MHC1.1	T	wt/KO/KO	G	G/T	G	G	wt/G12C	This work
	MHC2.1	T		G	G/T	G	G		
RKO KRAS G12V	MHV1.1	T	wt/KO/KO	G	G	G/T	G	wt/G12V	This work
	MHV2.2	T		G	G	G/T	G		
RKO KRAS G12D	MHD2.1	T	wt/KO/KO	G	G	G	G/A	wt/G12D	This work
	MHD1.1	T		G	G	G	G/A		

### Metabolome analyses

For the metabolome analysis, six biological replicates were investigated per cell mutant and QC were prepared and separately extracted to represent an average of all cell lines. To extract metabolites, 1 × 10^7^ cells were washed three times with ice cold PBS pH 7.4 and residual buffer was removed under vacuum and 3.5 ml HPLC-grade MeOH added at -20 °C. Cells were detached with a rubber-tipped cell scraper, transferred into polypropylene tubes and stored at -80 °C until metabolite extraction. HPLC-grade CHCl_3_ and milliQ-H_2_O was added to the sample (4:4:2.85/sample solution: CHCl_3_: H_2_O; fixed volume ratio), in this case 356 µl water was added to a 500 µl sample, followed by 500 µl chloroform. Tubes were shaken at 4 °C for 20 min, 1,400 rpm (Thermomixer) followed by centrifugation at 4 °C for 5 min at 16,100 g. The aqueous phase solvent was removed under reduced pressure in a vacuum concentrator and the residue dissolved in 5 % v/v acetonitrile solution and stored at -20 °C before UPLC-MS analysis. To reduce variation between sample groups due to retention time drift, column conditioning, or analyte carryover, the samples were randomized prior to analysis. The metabolite extracts were analyzed using a Waters Synapt G2-S ES-TOF LC-MS system equipped with a C18 column and a 17 min gradient. The RAW files obtained were converted to netCDF files using Databridge (MassLynx). Pairwise comparisons between sample sets was carried out using the XCMS software package [25] in RStudio to align mass chromatograms and identify peaks, which differ significantly between the two datasets and produce feature tables for further data analyses and visualizations.

### Metabolite Identification

The XCMS software package [25] was used to perform peak identification, retention time correction, and peak integration yielding a list of identified peaks by increasing *p*-value, which were then corrected for multiple comparisons (FDR). Identification of the features was performed by comparing their *m/z* ratios to a list of exact masses for known human endogenous metabolites from the human metabolome database (HMDB) with a mass spectrometric accuracy < 10 ppm.

### Metabolic Pathway Analysis

The mummichog2 software package [26] was used to identify metabolic pathways which differed significantly between mutants and their corresponding controls. The pathway analysis was performed through integration of both negative mode and positive mode mass spectrometric analysis to ensure high metabolite pathway coverage. The results from both analytical modes were combined using Fisher’s method to find the *Χ*^*2*^ value for each pathway, which was then converted into a *z*-value.

$$ {X}_4^2\sim -2\ln \left({p}_{+}\right)-2\ln \left({p}_{-}\right) $$

## Results

### Generation of isogenic cell models of ***KRAS*** and ***BRAF*** mutations

To better understand whether *KRAS* and *BRAF* mutations prevalent in CRC engender the same phenotype, we created a set of isogenic cell lines by introducing different mutant *KRAS* alleles in a genetic background where the mutant *BRAF* V600E allele had been removed by knock-out. Parental RKO cells [27] have two *BRAF* V600E mutant alleles and one wild-type allele, where the two *BRAF* V600E mutant alleles have been removed in RKO *BRAF* WT cells [5] whereas parental HCT116 cells [28] have a single *KRAS* G13D allele removed in HCT116 *KRAS* WT cells [24]. We used genome editing by recombinant adeno-associated virus (rAAV) technology [29] to knock in mutant *KRAS* and *BRAF* alleles in RKO *BRAF* WT and HCT116 *KRAS* WT cells, respectively. Gene targeting constructs were generated by amplifying homology arms from the respective targeted CRC cells followed by introduction the mutations by overlapping PCR [30] (Supplementary Fig. 1 A, Additional File 1). The *KRAS* mutant constructs were then used to target RKO *BRAF* WT cells, resulting in three independent edited clones of each *KRAS* genotype (Table 1, Supplementary Fig. 1B, Additional File 1). Presence of the desired *KRAS* mutation in targeted cells was demonstrated by sequencing the targeted *KRAS* exon and the expression of wild type and mutant alleles of *KRAS* and *BRAF* was confirmed by Sanger sequencing of the RT-PCR products (Supplementary Fig. 2 A-B, Additional File 1; Supplementary Tables 1, Additional File 2). Finally, expression of the desired wild-type and mutant transcripts of *KRAS* and *BRAF* genes was confirmed by transcriptome sequencing (Supplementary Fig. 3 A-G, Additional File 1). Thus, a set of cell models where *BRAF* V600E and the *KRAS* codon 12 and 13 mutations can be studied in the same genetic background was generated and validated.

### Transcriptomic, proteomic and metabolomic analyses of ***KRAS*** and ***BRAF*** mutations

**Fig. 1:**
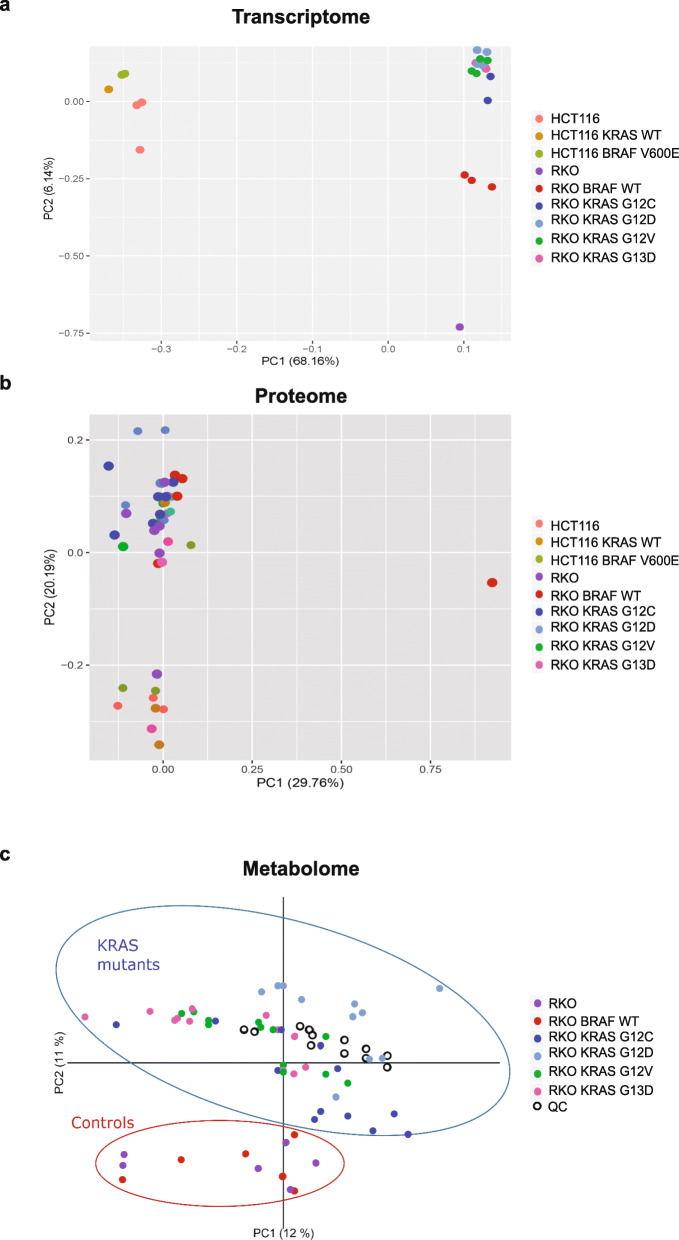
Integrative analysis of transcriptomes, proteomes and metabolomes of KRAS G12C/D/V and G13D mutant RKO colorectal cancer cells. **a** Principal Component Analysis (PCA) of transcriptome sequencing data  from ~25M reads/sample. **b** global proteomic data from ~4,500 proteins. **c** LC/MS metabolomics data from average 700 metabolites from knock-ins of different mutant KRAS alleles in RKO CRC cells deprived of their mutant BRAF allele.

To find and understand common and distinct phenotypes of different Ras pathway mutations in CRC, we characterized the transcriptomes, proteomes and metabolomes of RKO and HCT116 cells with *BRAF* V600E mutation, *KRAS* mutations, or no Ras pathway mutation. At the transcriptome level, principal component analysis (PCA) of differentially expressed transcripts in the RNA sequencing data showed clear separation by genetic background between HCT116 and RKO cell lines and the derived cell clones. Surprisingly, no clear separation by Ras pathway mutation was observed (Fig. 1a). Similarly, PCA analysis of ~ 4,500 detected proteins separated the cell clones by genetic background but not by *KRAS/BRAF* mutation status (Fig. 1b). However, the metabolomics PCA analysis separated RKO as well as HCT116 cell clones by their Ras pathway genotype (Fig. 1c). The *KRAS* mutant cell lines clustered by mutation, separating mainly along the first principal component, which supports that the introduced mutations are responsible for more variation than any other variable. Taken together, under normal cell culture conditions the overall impact of the Ras pathway mutations at the transcriptome and proteome levels appeared limited whereas stronger effects were observed at the metabolome level.

### Differential expression of genes and proteins in KRAS/BRAF mutant cells

**Fig. 2:**
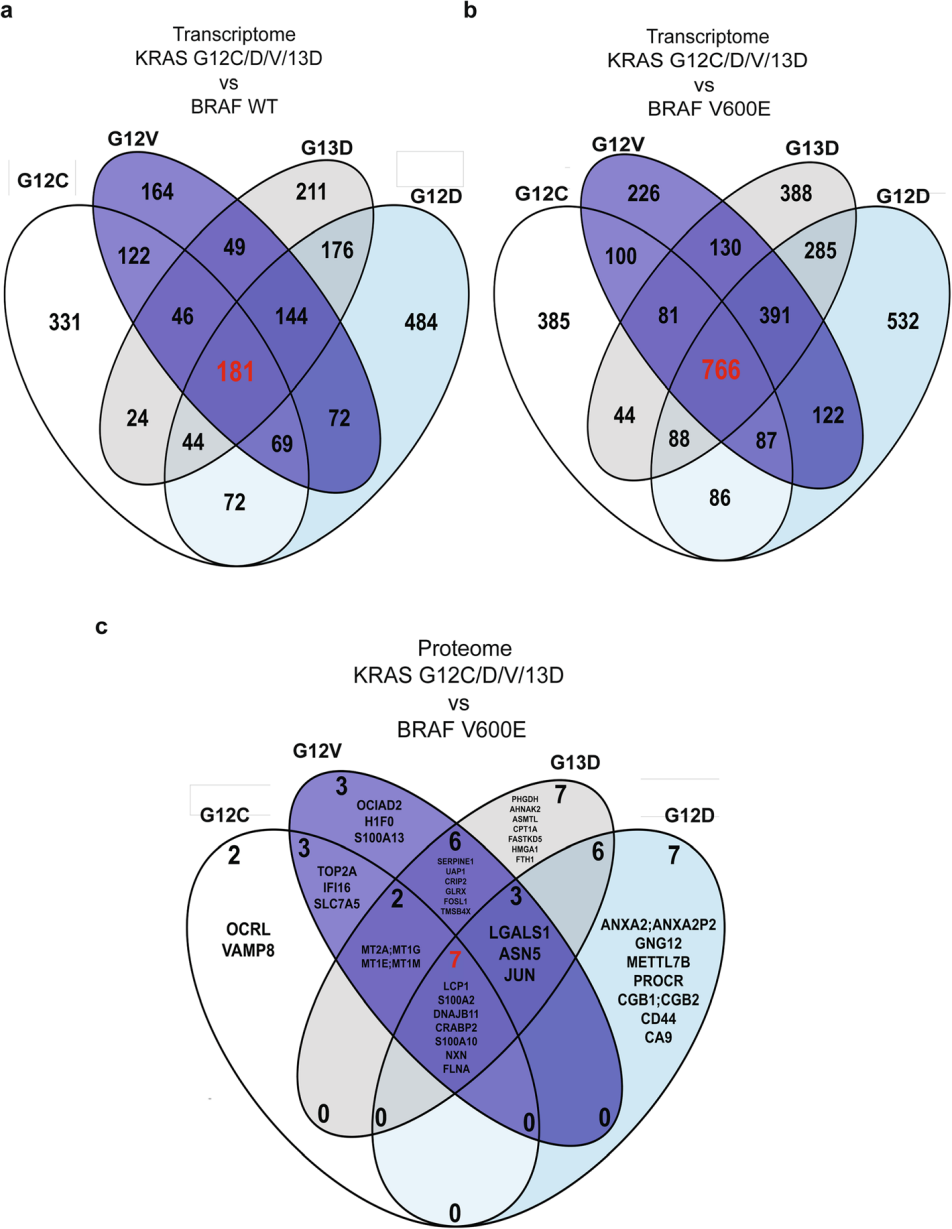
Transcriptomic and proteomic analyses reveal joint and mutation-specific regulation of gene and protein expression by KRAS G12C/D/V and G13D mutations. **a** and **b** Differentially expressed genes. **c** Differentially expressed proteins. All having ½log2 FC½ > 1 and adjusted *P* < 0.05 between A KRAS mutant and WT or b and c BRAF V600E RKO cells.

We next sought to determine whether the different *KRAS* and *BRAF* mutations alter the expression of specific genes or proteins. Because of the availability of multiple independent clones per genotype, we focused these analyses on the RKO genetic background. To find differentially expressed genes (DEGs) and proteins (DEPs) regulated by any or all of the different Ras pathway mutations, we compared their gene and protein expression data to the corresponding RKO isogenic controls (Supplementary Fig. 4 A-E and 6 A-D, Additional File 1; Supplementary table 2 A-J and 4 A-I, Additional File 2). We first identified 181 and 766 DEGs between all *KRAS* mutant clones and WT or *BRAF* V600E cells, respectively (Fig. 2a-b). For each *KRAS* mutation studied, the ratio of DEGs between comparisons to *BRAF* V600E or wildtype was in the range 1.90–2.49 (Supplementary Tables 2, Additional File 2). The notion that the transcriptional response differs more between *KRAS* and *BRAF* mutant cells than between *KRAS* mutants and cells lacking Ras pathway mutation was supported by analyses of HCT116 cells, where the ratio was 1.56 (Supplementary Fig. 5, Additional File 1). We hypothesized that the 22–34 % of DEGs found both in comparisons with *BRAF* mutant and wild-type cells and having the same direction of expression change could be specific to a particular *KRAS* mutation (Supplementary Fig. 5 A-D and F, Additional File 1). We identified 35, 70, 26 and 39 such *KRAS* G12C/D/V/G13D mutation specific DEGs, respectively (Supplementary Fig. 5 A-D and F; Supplementary Fig. 5E, Additional File 1; Supplementary Tables 3, Additional File 2). We next sought to determine whether these DEGs were also controlled by *KRAS* or *BRAF* mutations in clinical samples. Several DEGs were differentially expressed with the same direction of gene regulation in a KRAS G12 specific manner also in TCGA COAD data, including BCHE, BEST3, EXO1 [31], FCER2, FGF19, GPM6A, HOTAIR [31], KCNIP3, NTSR1, PRKAA2, SMC4, TMEM71, and TUBAL3 (Supplementary table 4 A-J, Additional File 2) where EXO1 and HOTAIR have previously been linked to Ras signaling. The expression of EXO1 and SMC4 is regulated by the DREAM complex [32], and they interact during DNA replication in yeast. Collectively, common and *KRAS* mutation specific DEGs with known as well as previously unknown links to the Ras pathway were identified.

**Fig. 3:**
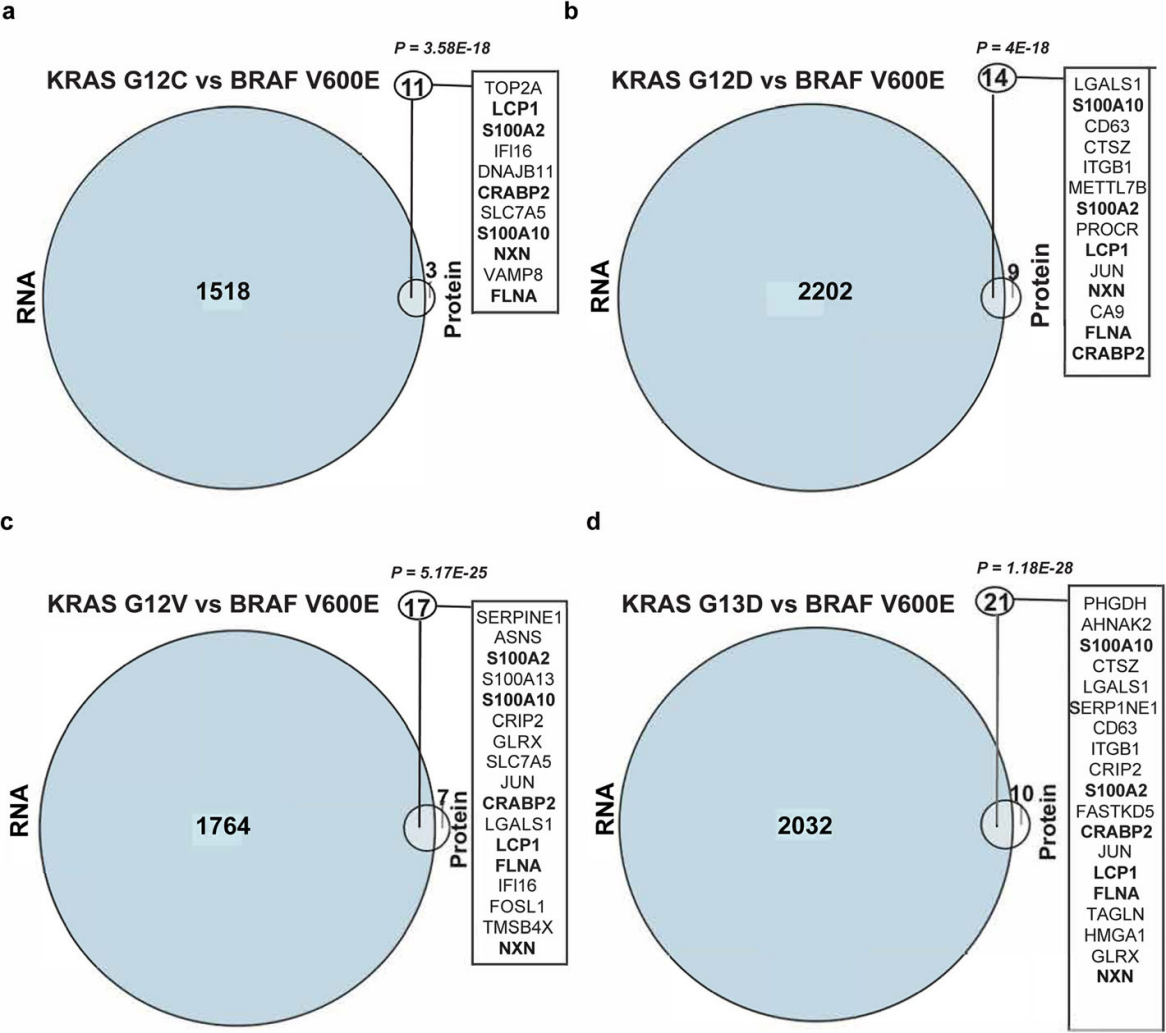
Differentially expressed proteins are primarily differentially expressed genes, but not vice versa, in KRAS mutants compared to BRAF V600E cells. Differentially expressed proteins (DEPs) were intersected with   differentially expressed genes from RNA sequencing data, comparing isogenic RKO BRAF V600E cells with KRAS G12C (**a**), G12D (**b**), G12V (**c**) and G13D (**d**). Differential expression was defined as ½log2 FC½ > 1 and adjusted *P* < 0.05 (hypergeometric distribution). Intersecting DEGs and DEPs are listed with genes common to all four comparisons in bold.

Next, we proceeded to identify DEPs regulated by Ras pathway mutations. The ratio of DEPs identified in comparisons of KRAS mutants to *BRAF* V600E or wild-type was in the range 7.4–24 (Supplementary Tables 5, Additional File 2). As compared to RKO cells with no Ras pathway mutation, known Ras regulated or interacting proteins were identified as DEPs: LGALS1 [33] was a DEP in *KRAS* G12D and G13D mutants, whereas IFI16 [34], S100A10 [35], CD44 [36], GLRX [37] and AHNAK2 [38] were DEPs in one of the *KRAS* mutants (Supplementary table 5B, D, F and H, Additional File 2). From the proteins highlighted in [7], AKAP12 was a DEG in comparisons of *KRAS* mutant to *BRAF* V600E as well as wild-type cells but not a DEP. Interestingly, 6 DEPs were common to all four *KRAS* mutations when comparing to *BRAF* V600E, of which 3 were upregulated (LCP1, S100A10 and S100A2 [39]) and 3 downregulated (CRABP2 [40], FLNA [38] and NXN) more than 10-fold in *KRAS* mutant clones (Fig. 2c) (Supplementary Fig. 6 A-D, Additional File 1; Supplementary table 5 A, C, E and G, Additional File 2). When identifying *KRAS* G12C/D/V/13D mutation specific DEPs versus *BRAF* V600E, we identified 2 (OCRL and VAMP8), 3 (OCIAD2, H1-0 and S100A13), 7 (ANXA2 [41], GNG12, METTL7B, PROCR, CGB1, CD44 and CA9) and 7 (PHGDH [42], AHNAK2 [38], ASMTL, CPT1A, FASTKD5, HMGA1 [43] and FTH1), respectively. Notably, CD44, PROCR and HMGA1 have previously been found upregulated by *KRAS* G12V [44]. To assess the link between transcription and translation, we identified 11, 14, 17 and 21 DEGs, respectively, as regulated at both levels by *KRAS* G12C/D/V/G13D mutation as compared to *BRAF* V600E (*p* ≤ 3.58E-18, hypergeometric distribution; Fig. 3a-d). Of these, CRABP2, FLNA, LCP1, NXN, S100A2, and S100A10 were regulated at both levels in *KRAS* mutant cells. While the majority (61–78 %) of DEPs in all four mutants were also DEGs, only 0.2–0.5 % of the DEGs were also DEPs; the vast majority of transcriptional regulation was not reflected in altered protein expression while more than half of DEPs were regulated through altered gene expression. Thus, 6/6 of DEPs identified in comparisons of mutant *KRAS* clones to isogenic cells with no Ras pathway mutation have previously known roles in Ras signaling, whereas 6 common and 19 *KRAS* mutation specific DEPs of which 8 were previously known were identified in comparison to *BRAF* V600E isogenic cells.

### Pathway analysis of transcriptome and proteome datasets

**Fig. 4:**
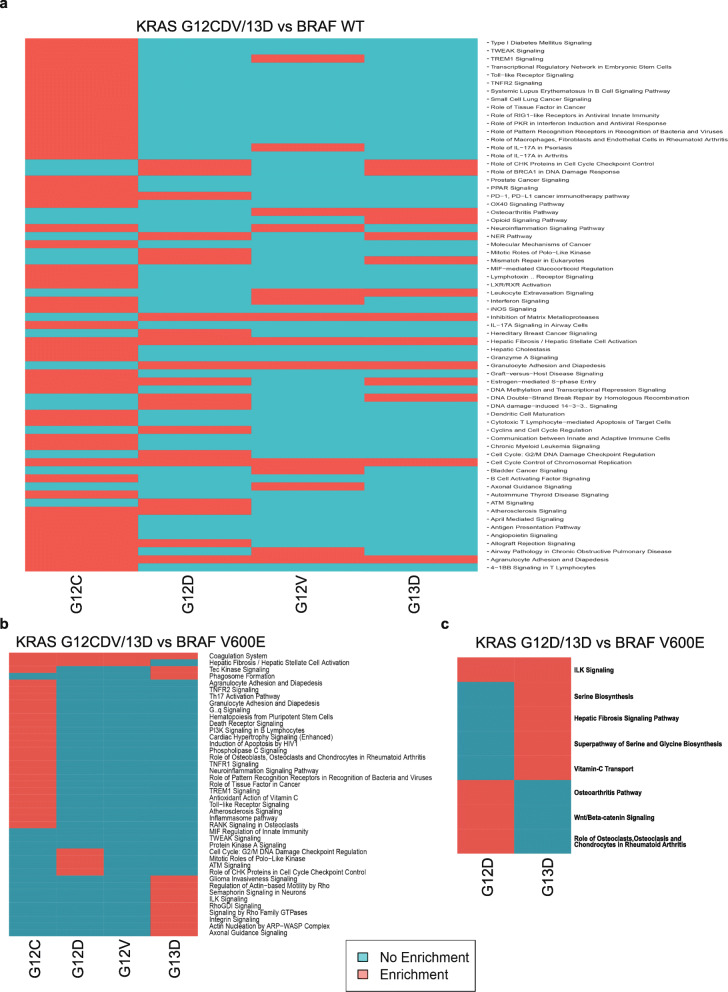
Joint and mutation specific regulation of molecular pathways by KRAS mutations. Binary heatmaps from Ingenuity Pathway Analysis (IPA) of KRAS mutant cells compared to wild-type or BRAF V600E mutant cells. IPA analysis of transcriptome comparisons of (**a**) KRAS G12C/D/V/G13D versus wild-type, (**b**) KRAS G12C/D/V and G13D vs BRAF V600E. Proteome DEPs from (**c**) KRAS G12D/13D vs BRAF V600E comparisons; only these two comparisons had sufficient differentially expressed proteins (DEPs) for IPA analysis. The IPA analysis included DEGs or DEPs fulfillingㅣlog2 FCㅣ > 1, adjusted *P* value <0.05 and Z-score ≥ 2 or ≤ - 2.

Next, we sought to investigate common and unique pathways controlled by different *KRAS* mutations and whether different pathways are involved in *KRAS* and *BRAF* signaling through Ingenuity Pathway Analysis (IPA) of transcriptomes (Supplementary table 6 A-D, Additional File 2) and proteomes (Supplementary table 7 A-B, Additional File 2). In comparison to cells lacking Ras pathway mutation, BRCA1-DNA damage signaling, mismatch repair, and cell-cycle checkpoint signaling was enriched in *KRAS* G12D mutant cells (Supplementary table 6B, Additional File 2) and actin-based motility, RhoGTPase signaling, and axonal guidance signaling in *KRAS* G13D (Supplementary table 6D, Additional File 2) (Fig. 4a). Compared to *BRAF* V600E cells, the Integrin-Linked Kinase (ILK) pathway was significantly altered in *KRAS* G13D cells at the transcriptome and protein levels, as well as in *KRAS* G12D at the proteome level (Fig. 4b and c; Supplementary table 6D and 7B, Additional File 2). The Molecule Activation Prediction (MAP) tool predicted ILK signal activation based on the protein expression changes in *KRAS* G12D and G13D, which is in turn predicted to activate downstream genes related to cell proliferation, adhesion, motility, cancer progression EMT, cancer stem cell markers and chemoresistance [45, 46] (Supplementary Fig. 7 A-C, Additional File 1). Additionally, a canonical pathway analysis of DEPs versus *BRAF* V600E showed enrichment of the Wnt/beta-catenin signaling pathway in *KRAS* G13D clones and of the serine biosynthetic pathway in G12D clones (Supplementary table 7 A-B, Additional File 2). Taken together, known (MMR, ILK, cell cycle checkpoint, actin-based motility, RhoGTPase signaling, axonal guidance, serine and glycine biosynthetic pathways) as well as novel (BRCA1-DNA) pathways were significantly regulated as a consequence of Ras mutation and the ILK pathway emerged as regulated by *KRAS* but not *BRAF* mutation.

### Differential expression of immune related genes by Ras pathway mutations

Co-expression of immune related genes and pathways has been used for immunological stratification of CRC [47, 48]. Several different immune regulatory pathways such as IFNα, IFNγ, host-defense and antigen presentation are suppressed in *KRAS* mutated CRC [49]. This prompted us to investigate the expression status of genes of three pathways in the different isogenic cell models: (i) the IFNα pathway (105 genes), (ii) the IFNγ pathway (149 genes), and (iii) *KRAS* mutually exclusive genes (56 genes) as defined in [49]. Several genes from these pathways were differentially expressed here in all the *KRAS* mutants (Supplementary Fig. 8 A-C; Supplementary table 8 A-C). These included BATF2, CCRL2, IFI27, IFI44L, SAMD9L, and TRIM21 from the IFNα pathway, and IRF9, MX2, and HLA-B from the IFNγ pathway which were DEGs in all the *KRAS* mutant cell lines when compared to wild-type and *BRAF* V600E cells (Supplementary Fig. 8 A-B, Additional File 1; Supplementary table 8 A-B, Additional File 2). Interestingly, immune genes mutually exclusive to *KRAS* (e.g., *BCHE*, *CCSER1*, *DDX60L*) were also significantly differentially expressed in the transcriptome of cell lines with different *KRAS* mutations as compared to *BRAF* V600E mutants (Supplementary Fig. 8 C, Additional File 1; Supplementary Table 8 C, Additional File 2). A subset of immune genes was expressed in a mutation specific manner, such as B2M (KRAS G12C), IFIT3 (G12D) and IL4R (G13D) in the IFNα pathway, IFIT3 (G12D), PARP14 (G12C), PTGS2 (G12V) in the IFNγ pathway, and ENPP6 (G12V), ARSD and IRF2BPL (G13D) genes with genomic deletions mutually exclusive to *KRAS* mutations were differentially expressed as compared to either *BRAF* V600E mutant or *BRAF* WT (Supplementary Fig. 8 A-C, Additional File 1; Supplementary table 8 A-C, Additional File 2). Moreover, some of the immune related genes such as IFI27, IFI35 (IFNα pathway), IFITM2, OGFR (IFNγ pathway) and CCSER1 (mutually exclusive to *KRAS*) were found to be differentially regulated in both RKO KRAS G13D vs. RKO as well as HCT116 BRAF V600E vs. HCT116 comparisons (Supplementary Fig. 8 A-C, Additional File 1; Supplementary table 8 A-C, Additional File 2). Taken together, 70 % of IFNα pathway genes, 63 % of IFNγ pathway genes, and 50 % of *KRAS* mutually exclusive genes were significantly differentially expressed in the *KRAS* mutant cell lines (*p* ≤ 2.94E-12, hypergeometric distribution) [47–49].

### Altered cellular metabolism as a consequence of Ras pathway mutations

**Fig. 5:**
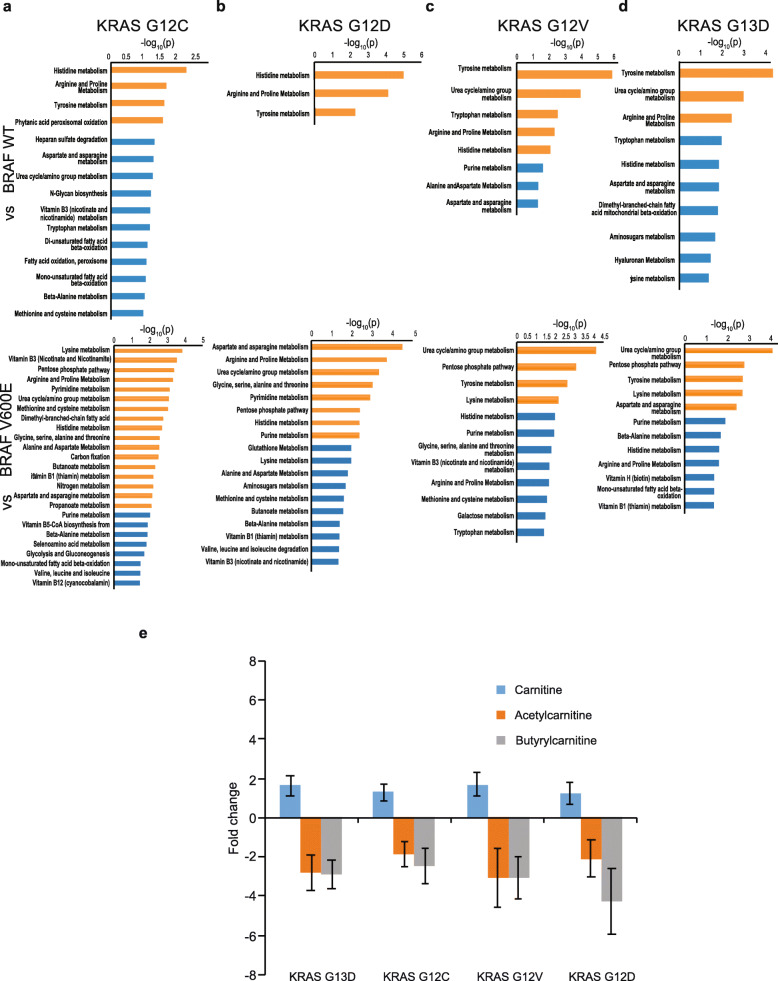
Metabolic pathway analysis reveals regulation of amino acid metabolism and carnitine biosynthesis by Ras pathway mutations. LC-MS metabolomics analysis was performed on KRAS knock-ins and isogenic controls followed by pathway analysis comparing (**a**) G12C, (**b**) G12D, (**c**) G12V and (**d**) G13D vs BRAF V600E (upper panel) and wildtype (lower panel). Pathways with *P* < 0.001 and 0.05 were designated with orange and blue bars, respectively. **e** The KRAS mutants showed increases in free Carnitine and decreases in acetyl and butyrylcarnitines compared to the isogenic BRAF wild-type control cell line. Average values were from six biological replicates with SD.

To understand how different *KRAS* mutations or the *BRAF* V600E mutation alter cellular metabolism, we performed UPLC-MS based metabolomic analysis. Whereas the PCAs comparing biological replicates demonstrated that the G12C cell clones exhibited separation among them, no significant differences between the two independent clones with *KRAS* G12D, G12V or G13D mutations were observed (Supplementary Fig. 9 A-D, Additional File 1). Pairwise comparisons revealed much fewer significantly altered metabolites and pathways (0–3), confirming that the independent clones were near identical in their metabolism. However, significant differences were observed between the mutant clones and their isogenic controls (Fig. 1c, Supplementary Fig. 9E, Additional File 1). Altogether, the clear separation of Ras pathway mutant and control cells into discrete clusters in the PCA analysis was a strong indication that *KRAS* and *BRAF* mutations alter the metabolome. In a metabolic pathway analysis, where an average of 413 significant features were assigned to compounds in positive mode, and 303 in negative mode with FDR < 0.01, altered amino acid metabolism was observed following Ras pathway activation (Fig. 5a-d, Supplementary Fig. 10 A-B, Additional File 1 and Supplementary table 9 A-J, Additional File 2). Across all mutants, we observed significant alterations in the tyrosine, histidine, arginine and proline metabolic pathways relative to their respective isogenic controls (Fig. 5a-d; Supplementary Fig. 10 A-B, Additional File 1 and Supplementary table 9 A-J, Additional File 2) in agreement with previous studies [8, 50, 51]. Furthermore, certain amino acid metabolic pathways were significantly altered in a mutation specific manner, such as lysine metabolism in *KRAS* G12C and G12V (Fig. 5b and Supplementary Fig. 10 A-B, Additional File 1; Supplementary Table 9 C-D and 9I-J, Additional File 2) and fatty acid oxidation in *KRAS* G12C and G13D in comparison to their respective isogenic controls (Fig. 5a and d; Supplementary table 9 A-B and 9G-H, Additional File 2). Additionally, the urea cycle/amino group, purine, pyrimidine and pentose-phosphate metabolic pathways were significantly altered by *KRAS* or *BRAF* mutation (Fig. 5a-d and Supplementary Fig. 10 A-B, Additional File 1; Supplementary table 9 A-H, Additional File 2). Interestingly, genes related to amino acid metabolism and fatty acid metabolism extracted from Kyoto Encyclopedia of Genes and Genomes (KEGG) database were significantly enriched in the transcriptome data (Supplementary Fig. 11 A-J, Additional File 1; Supplementary table 10 A-J, Additional File 2). Key metabolic pathway genes such as GOT1, which has roles in more than one metabolic pathway (i.e., tyrosine, histidine and proline metabolism) were differentially expressed in all *KRAS* mutants (Supplementary Fig. 11 A, C and E, Additional File 1). Taken together, the *KRAS* and *BRAF* mutations led to similar metabolic consequences, primarily affecting pathways connected to amino acids metabolism and the urea cycle/amino group metabolisms.

### Carnitine metabolism is regulated by ***KRAS*** and ***BRAF*** mutations

In the metabolomics analysis, significant alterations of L-carnitine derivatives were observed (Fig. 5e; supplementary Fig. 12, Additional File 1). L-Carnitine is acylated by fatty acids in the cytosol and then transported into the mitochondria, where the fatty acid is released for *β*-oxidation. Carnitine is transferred back to the cytosol after release of fatty acids. Hence, carnitine plays an important role in fatty acid oxidation and mitochondrial metabolism [52]. Here, we observed higher abundance of free carnitine in *KRAS* mutant RKO cells, while its esters such as acetyl- and butyryl-carnitine were less abundant (Fig. 5e). Interestingly, the majority of all detected acylated carnitines were decreased in *KRAS* and *BRAF* mutant clones whereas medium-chain and long-chain carnitines such as tetradecanoyl, docanoyl and octanoyl carnitine were more abundant (Supplementary Fig. 12, Additional File 1). Furthermore, differential expression of carnitine biosynthesis related genes such as CPT1 and 2 was evident in *KRAS* mutants compared to *BRAF* V600E (Supplementary table 2 A, C and E, Additional File 2). Members of the SLC22 transporter family, such as *SLC22A5*, play important roles in carnitine transport [52, 53] and mutation in *SLC22A5* has been implicated in systemic carnitine deficiency related cardiomyopathy, skeletal myopathy and metabolic abnormalities [54]. Another member, *SLC22A4*, has been implicated in carnitine transport [53] and was differentially expressed in *KRAS* G12D/V versus wild-type cells (Supplementary Table 2 C and E, Additional File 2). Notably, several uncharacterized members of this transporter family were differentially expressed here (*SLC22A15* in *KRAS* G12D/V vs. *BRAF* WT and *KRAS* G13D vs. *BRAF* V600E (Supplementary table 2D, F and G, Additional File 2), *SLC22A17* in HCT116 BRAF V600E vs. HCT116 (Supplementary table 2I, Additional File 2), *SLC22A18* in RKO KRAS G12C/D/V vs. RKO BRAF WT (Supplementary table 2B, D and F, Additional File 2) and *SLC22A*23 in RKO KRAS G12C/D/V/13D vs. RKO (Supplementary table 2 A, C, E and G, Additional File 2) [53]. Thus, levels of carnitines and their biosynthetic genes were differentially regulated by mutant *KRAS* and *BRAF*.

## Discussion

Even though KRAS and BRAF act in the same pathway, as evidenced by the mutual exclusivity of somatic mutations in CRC, different clinico-pathological phenotypes have been ascribed to different *KRAS* and *BRAF* mutations. Here, we sought to understand the contributions of, and differences between, prevalent mutations in *KRAS* and *BRAF* in CRC. Previous biochemical and structural studies have shown that amino acids 12 and 13 are located in the P-loop of KRAS. The P-loop is adjacent to the Switch-II region, and its position determines the binding of GAPs as well as interactions with downstream signaling molecules such as RAF1, PI3K and Ral [55]. If the side chain of the mutated amino acid is bulky (e.g. KRAS G12D/V), it will displace the Switch-II region which, in turn, would (i) decrease the GTPase activity as well as (ii) bind to different downstream signalling molecules [56, 57]. It is therefore plausible that the different codon 12 and 13 mutations can result in joint as well as mutation specific downstream signalling, in turn leading to slightly different gene and protein expression patterns and clinical outcomes. Hence, we hypothesized that an integrative analysis of the transcriptome, proteome and metabolome alterations induced by *KRAS* and *BRAF* mutations in the same genetic background could improve the understanding of the effects of different *KRAS* mutations on signaling pathways, cellular functions, and in the extension clinical outcomes. Such an integrated -omics approach has been proposed to be a more robust means to identify driver genes and sub-stratify tumors [58–60].

At the overall transcriptome level, wild-type and *BRAF* V600E cells clustered separately from the *KRAS* mutants but the PCA showed little distinction between different *KRAS* mutants in RKO cells which is in line with [61]. Given that the studied Ras mutations alter two adjacent amino acids, their downstream effects would be expected to have much in common but potentially also some mutation specific patterns. Indeed, the DEGs unique to each *KRAS* mutation were limited to 26–70 genes. In non-small cell lung cancers, *KRAS* G12C/V activated the Ral pathway but reduced AKT phosphorylation, whereas *KRAS* G12D activated phosphatidylinositol 3-kinase (PI3K) and mitogen-activated protein/extracellular signal-regulated kinase kinase (MEK) signaling [57]. Hence, the mutation specific gene expression signatures of *KRAS* mutant cells might provide clues to the *KRAS* mutant specific clinical outcomes. Interestingly, we identified the greatest number of DEGs in the *KRAS* G12D mutant, which also has the highest mutation prevalence (29 %) in CRC [62]. Furthermore, 116 DEGs identified here were differentially expressed with the same trend of expression in RNAseq data from *KRAS* mutant TCGA CRC tumors, strengthening the relevance of these specific genes. At the global proteome level, the 6 DEPs in *KRAS* mutants versus *BRAF* V600E cells (LCP1, S100A2, CRABP2, S100A10, NXN and FLNA) had all previously been linked to Ras signaling, effectively demonstrating that the combination of isogenic cell models of mutations coupled with global proteomics used here can accurately identify Ras pathway regulated proteins. Of these, S100A2 has been implicated in CRC [63, 64] as a prognostic marker [65], and S100A10, itself a biomarker in CRC [66] was shown to play a pivotal role in gastric cancer invasion [67]. Hence, we reasoned that the genes differentially expressed at both transcript and protein level would be of highest importance [68].

Intriguingly, the number of DEPs identified in the comparison between *KRAS* and *BRAF* mutant cells was 4-fold higher than between *KRAS* mutant and cells lacking Ras pathway mutation. The larger transcriptome and proteome differences observed between *KRAS* and *BRAF* mutants than between *KRAS* mutants and cells lacking Ras pathway mutation are in line with previous findings in transcriptomes of 381 human CRCs with *BRAF*, *KRAS* or unknown mutation in the EGFR pathway, where the *BRAF* mutant tumours constituted a separate group [69]. But how can this difference be explained? Apart from BRAF, active KRAS can impinge on several pathways including the PI3K and Ral pathways. Here, of known Ral pathway target genes [70], PLEKHA4 was ~ 40-fold upregulated in all *KRAS* mutants, PGF in G12D and G13D mutants, and HOXA6 in G12C and G13D mutants versus *BRAF* at the transcript level. The PI3K pathway member MRAS was differentially expressed at the transcript level in all *KRAS* mutants compared to wild-type cells. Furthermore, the 11, 14, 17 and 21 genes regulated both at the transcript and protein level in the KRAS G12C/D/V/13D mutant cells, respectively, as compared to *BRAF* V600E deserve further functional investigation as to whether they can help explain the clinically observed differences between *KRAS* and *BRAF* mutant CRCs.

Given the observed differences at the transcriptome and proteome level, one could expect differential pathway regulation when comparing *KRAS* mutant to wild-type and *BRAF* V600E cells. Indeed, different pathways emerged as significantly regulated between the *KRAS* mutants as well as between *KRAS* and *BRAF* mutants at the transcript level and between *KRAS* G12D/13D and *BRAF* mutants at the protein level. We deemed pathways affected at both transcriptome and proteome level as of particular interest, and the ILK pathway emerged as such in comparing *KRAS* to *BRAF* mutant cells. The ILK pathway has been associated with migration, invasion, EMT, cancer stem cell marker and chemotherapy resistance in CRC [45, 71]. Furthermore, the ILK pathway was found to cross-talk with the KRAS pathway via a KRAS-ILK-hnRNPA1 regulatory loop in pancreatic cancer [72] as well as in lung adenocarcinoma [73]. In prostate cancer, KRAS regulates ILK expression mediated by E2F1 in a KRAS-E2F1-ILK-hnRNP1 loop [72]. As there are at least 14 putative E2F1 binding sites in the upstream promoter region of ILK, this would be a plausible mechanism for the regulation of ILK by KRAS in CRC observed here. Taken together, these findings support that ILK signaling is regulated by *KRAS* but not *BRAF* mutations through E2F1.

Previous studies based on expression profiling of CRC demonstrated that tumors with *KRAS* mutations had poor infiltration of T cells and high expression of inhibitory molecules (i.e. CTLA4, PDL1, PDL2, LAG3 and TIM3) [47]. Further, *KRAS* mutation suppressed the IFNγ pathway in human CRC and reduced immune infiltration [48]. Recently, KRAS-IRF2 mediated immune suppression and immune therapy resistance was demonstrated in CRC [49]. This prompted us to investigate the status of immune related genes in our datasets from different *KRAS* and *BRAF* mutant isogenic cells. Indeed, 70 % of IFNα pathway, 63 % of the IFNγ pathway and 50 % of *KRAS* mutually exclusive genes were significantly differentially expressed in *KRAS* mutant cell lines as compared to either *BRAF* V600E mutant or wild type isogenic control cell lines. These differential expression signatures of immune related genes can help explain the different immune landscapes between *KRAS* and BRAF V600E [74].

Oncogenic *KRAS* plays important roles in the metabolic rewiring of cancer cells, and has been implicated in the decoupling of glycolysis and TCA metabolism [50]. Here, the metabolic pathways of the amino acids histidine, tyrosine, arginine, and proline was altered in all *KRAS* mutants as compared to cells lacking Ras pathway mutation in agreement with [75]. While *KRAS* G12D had the fewest altered metabolic pathways, restricted to said amino acids, *KRAS* G12C was identified with most altered pathways, which is in line with previous studies as well [8, 75]. Interestingly, the differences between *KRAS* mutants and *BRAF* V600E were more striking than comparisons to wild-type cells also at the metabolome level, with 1.2-6-fold more regulated metabolic pathways. These differences were partially overlapping, and affected arginine, histidine, and proline metabolism, but also included a range of other amino acid synthesis pathways, the pentose phosphate pathway, purine and pyrimidine biosynthesis, vitamin B metabolism and other pathways. Interestingly, PHGDH, the first committed enzyme in serine biosynthesis, was a DEP in *KRAS* mutant versus *BRAF* V600E RKO cells, which was reflected in the metabolic pathway analysis as altered serine metabolism and thus constitutes a direct link between proteome and metabolome alterations. Taken together, this suggests that parts of the metabolome regulation downstream of mutant *KRAS* isoforms are independent of BRAF signaling, which opens up a new avenue for investigation identified in this study.

It is conceivable that metabolome alterations can result in biomarkers of Ras pathway activity or provide new targets for therapies. Here, the levels of carnitine derivatives were altered by activating *KRAS* or *BRAF* mutation, with higher abundance of free carnitine versus wild-type cells, while acetyl and butyrylcarnitines were less abundant. The majority of all detected acylated carnitines were decreased in *KRAS* and *BRAF* mutant clones whereas tetradecanoyl, docanoyl and octanoyl carnitines were more abundant. Carnitine is acylated by fatty acids in the cytosol, which it then transports into the mitochondria where the fatty acid is released and undergoes *β*-oxidation. The Carnitine is then transferred back to the cytosol to repeat the cycle. In this process, carnitine palmitoyltransferase 2 (CPT 2; EC 2.3.1.21) transfers long-chain fatty acids from the cytosol to the mitochondrial matrix for *β*-oxidation and constitutes the rate-limiting step in mitochondrial fatty acid oxidation [52]. Several lines of evidence support a link between the Ras pathway and carnitine metabolism: (i) the Costello syndrome, a RASopathy caused by constitutional activating mutations in HRAS, has been linked to impaired carnitine mediated fatty acid oxidation [76], (ii) the proliferation and tumorigenesis of *BRAF* V600E melanoma cells was decreased after CPT1A knockdown [77], (iii) oncogene-induced senescence was reported to be reverted by genetic inhibition of CPT1, [78], (iv) somatic mutation in *CPT1A* is mutually exclusive to somatic *KRAS* mutation in 4121 COAD samples (*P* < 0.001, Fisher Exact Test with q < 0.0001 from Benjamini-Hochberg FDR corrections; cBioportal). Here, gene expression of carnitine biosynthesis related genes such as CPT1 and CPT2 was significantly differentially regulated and CPT1A was a DEP. Etomoxir, an inhibitor of CPT1, was launched as a diabetic drug [79] and has undergone clinical trials for cardiovascular disease [80]. The growth of human bladder cancer cells was significantly inhibited by Etomoxir [81], but its use has been limited by hepatotoxicity [82]. Thus, regulation of carnitine metabolism by Ras pathway activation is worthy of further studies aiming at therapies targeting the pathway.

## Conclusions

To date, this study is the first to dissect the differences between different *KRAS* mutants and *BRAF* V600E using an integrative -omics approach applied to isogenic cell lines which were either *KRAS* or *BRAF* mutants in their parental form, modified by gene targeting, thereby preserving endogenous gene expression levels. The different clinico-pathological phenotypes of tumors with *KRAS* and *BRAF* mutations [2] may be explained by differential reprogramming of transcriptomes, proteomes and metabolomes. The limitations of this study include the use of a single genetic background for the majority of analyses. While the isogenic knock-in systems facilitate direct comparisons of different *KRAS* mutations, the findings could be further strengthened through observations in additional model systems. The dynamics of cell signaling as well as the *in vivo* response to EGFR inhibition as a function of Ras pathway mutations also merit further investigation. Here, we have confirmed known Ras pathway induced alterations and identified novel regulated genes, proteins, pathways and specific metabolites constituting a rich source for further studies. Knowledge of such alterations induced by the prevalent EGFR/Ras/MAPK pathway mutations in CRC can aid drug discovery aiming at personalized treatments as well as development of companion diagnostics.

## Supplementary Information

**Additional file 1:Supplementary Figure S1**. Knock-in of ***KRAS*** G12C/D/V/13D in human RKO colorectal cancer cells by homologous recombination.** A** An AAV gene targeting construct was designed to introduce the *KRAS G12C/D/V/13D* allele in the second exon of *KRAS* in RKO BRAF WT cells. Numbers indicate primers used for homology arm amplification (7–10) (Supplementary Tables 1, Additional File 2), PCR screening for construct integration (11 and 12) and Cre-mediated removal of the resistance marker (13 and 14) (Supplementary Tables 1, Additional File 2). **B** PCR detection of targeted *KRAS* alleles in RKO *BRAF WT* human colorectal cancer cells in three independent clones. The PCR products of 1181 and 1050 bp represents wild type and targeted alleles, respectively. **Supplementary Fig. 2. **Detection of knock-in alleles by Sanger sequencing. Sanger sequencing (Supplementary Table 1) was performed using cDNAs from two different clones of (**A**) RKO *KRAS* G12C/D/V/13D knock-ins as well as single clone of RKO BRAF WT isogenic control, and from single clone (**B**) HCT116 *BRAF* V600E knock-in and HCT116 KRAS WT isogenic control. **Supplementary Fig. 3. **Validation of expression ***KRAS*** knock-in alleles by transcriptome sequencing. Illumina-based transcriptome sequencing (~ 25 M reads/sample) using total RNAs from two different clones of (A) RKO KRAS WT control, (B-E) G12C/D/V and G13D knock-ins and from single clone (**F**) HCT116 KRAS WT isogenic control as well as (**G**) HCT116 *BRAF* V600E knock-in. Presence of knock-in alleles in the transcriptome datasets were visualized in IGV. **Supplementary Fig. 4. **Differentially Expressed Genes (DEGs) from transcriptomes of ***KRAS*** mutant cells. Heatmap representations of the top 100 normalized DEGs with ㅣlog_2_ FCㅣ > 1 and adjusted *P <* 0.05 from comparisons of KRAS mutations (**A**) G12C, (**B**) G12D, (**C**) G12V and (**D**) G13D to wildtype (upper panel) and BRAF V600E (lower panel) RKO cells were represented with heatmaps. Similarly, heatmaps of DEGs from comparisons of (**E**) HCT116 BRAF V600E vs. HCT116 KRAS G12D (upper panel) and HCT116 KRAS WT (lower panel). Transcriptome data was normalized using the EBseq R package (Leng and Kendziorski 2019). Both samples and genes were clustered by Euclidian distances and row-wise scaling was applied to the 100 DEGs with lowest adjusted *p*-values to generate the heatmaps. **Supplementary Fig. 5. **Mutation specific Differentially Expressed Genes in ***KRAS*** G12C/D/V/13D and BRAF V600E cells. To identify genes regulated by a specific Ras mutation, the DEGs from comparisons of KRAS mutant cells to wildtype and BRAF V600E cells were intersected and DEGs having the same direction of expression change (i.e. upregulation or downregulation in both wildtype and V600E comparisons) were selected (green field) for (**A**) G12C, (**B**) G12D, (**C**) G12V, (**D**) G13D, respectively. The KRAS mutation specific DEGs were represented in 4-way Venn diagram (**E**). Similarly, BRAF V600E specific DEGs were identified in HCT116 cells (**F**). **Supplementary Figure S6. **Differentially Expressed Proteins (DEPs) in ***KRAS*** versus ***BRAF*** mutant RKO cells. Top 50 DEPs with ㅣlog_2_ FCㅣ > 1 and adjusted *P <* 0.05 from the proteome datasets of the comparisons of (A) G12C, (B) G12D, (**C**) G12V and (**D**) G13D KRAS mutants to BRAF V600E cells were represented with the lowest adjusted *p*-values. Data from LC-MS based proteomic analysis after normalization using quantile normalization and log_2_ transformation. Both samples and genes were clustered following Euclidian distances and row-wise scaling was applied to 50 DEPs with lowest adjusted p-values to create the heatmaps. **Supplementary Figure S7. **The ILK signaling pathway is altered at both RNA and protein level by KRAS but not ***BRAF*** mutation. The ILK pathway was enriched in IPA analysis of DEGs and DEPs fulfilling ㅣlog_2_ FCㅣ > 1, adjusted *P <* 0.05 and Z-score ≥ 2 or ≤ -2 in transcriptome data from (**A**) *KRAS* G13D vs. *BRAF* V600E and proteomic dataset from (**B**) *KRAS* G12D vs. *BRAF* V600E and (**C**) *KRAS* G13D vs. *BRAF* V600E comparisons, respectively. The IPA overlay tool was used for overlaying the expression fold change values of the differential expression analysis followed by the analysis with IPA Molecular Activity Prediction (MAP) tool to predict *in silico* down-stream and up-stream activation or inhibition of molecules and/or processes (cell proliferation, adhesion, tumor angiogenesis and tissue invasion) based on the observed expression changes due to KRAS G12D/13D mutations. Pink, up-regulated genes; green, down-regulated genes. **Supplementary Figure S8. **Differentially expressed immune related genes. DEGs with ㅣlog_2_ FCㅣ > 1 and adjusted *P <* 0.05 from transcriptome data were related to the IFN-alpha pathway (A) or IFN-gamma pathway (B) and immune genes whose genomic deletions were mutually exclusive to *KRAS* mutations (C). Supplementary Fig. 9. Similarity of cell clone metabolism between duplicate cell clones with the same ***KRAS*** knock-in mutation. Principal Component Analysis (PCA) of the metabolome datasets from the two included clones of KRAS G12C (**A**), G12D (**B**), G12V (**C**), G13D (**D**) and one clone each of (**E**) HCT116 KRAS WT and HCT116 BRAF V600E. **Supplementary Figure S10. **Amino acid metabolism is altered by ***KRAS*** and ***BRAF*** mutation in HCT116 cells. LC-MS based metabolomic analyses were performed using BRAF V600E knock-ins and isogenic control cells and subjected to pathway analysis comparing BRAF V600E vs. KRAS G12D (**A**), BRAF V600E vs. KRAS WT (**B**) and isogenic controls (**C**) cells. Pathways with *P* < 0.001 and 0.05 were designated with orange and blue bars, respectively. **Supplementary Figure S11. **Metabolic pathway genes are deregulated in the transcriptomes of ***KRAS*** and ***BRAF*** V600E mutant cells. The enzymes for the substrate or product metabolites identified were searched in the KEGG database, and the genes encoding enzymes which belong to the respective pathway were extracted. The extracted genes were analyzed in the transcriptome data for their Differential Gene Expressions (DEGs) with log2 > 1 and adjusted *P value < 0.05* from comparisons among RKO KRAS G12C/D/V/13D vs. RKO BRAF V600E (**A, C, E, G**), RKO BRAF wild-type (**B, D, F, H**), HCT116 BRAF V600E vs. HCT116 KRAS G13D (**I**) and HCT116 BRAF V600E vs. HCT116 KRAS wild-type (**J**). **Supplementary Figure S12. **Carnitine levels in knock-ins of ***KRAS*** G12C/D/V/13D. The majority of all detected acylated carnitines were decreased in *KRAS* and *BRAF* mutant clones whereas tetradecanoyl, docanoyl and octanoyl carnitine were more abundant.

**Additional file 2:Supplementary Table 1. **. Primers used for isogenic cell model generation and validation. List of primers for generating isogenic cell models for different KRAS and BRAF mutations using rAAV mediated genome editing (2 A). Primers for validations by Sanger sequencing for presence of mutations of interest of KRAS and BRAF (2B) and the validation results (2 C). **Supplementary Table 2. **Differentially Expressed Genes (DEGs) in ***KRAS*** and ***BRAF*** isogenic cell models. Differentially expressed genes (DEGs) with ㅣlog_2_ FCㅣ > 1 and FDR ≤ 0.05 from comparisons of RKO cells with *KRAS* G12C/D/V/G13D to wildtype (2 A, C, E, G) or *BRAF* V600E (2B, D, F, H) and HCT116 BRAF V600E to HCT116 (2I) or HCT116 KRAS WT (2 J). FDR, False Discovery Rate; log_2_FC, log2 transformed fold change of gene expression. **Supplementary Table 3. **Differentially Expressed Genes (DEGs) from overlapped Venn regions (Supplementary Fig. 5 A-D and F) and their trend of expression in the transcriptome datasets with logFC values. Differentially expressed genes (DEGs) with ㅣlog_2_ FCㅣ > 1 and FDR ≤ 0.05 from comparisons of RKO cells with *KRAS* G12C/D/V and G13D to wildtype or *BRAF* V600E were identified in the overlapped regions of the Venn diagramme analysis (Supplementary Fig. 5 A-D and F, Additional File 1).The “Trend of expressions” were determined as all negative, negative log_2_FC value in both of the comparisons; all positive, positive log_2_FC value in both of the comparisons and mixed, negative and positive log_2_FC value in one or another. Here, FDR, False Discovery Rate; log_2_FC, log2 transformed fold change of gene expression. **Supplementary Table 4. E**xpression status of Differentially Expressed Genes (DEGs) from ***KRAS*** and ***BRAF*** isogenic cell models in TCGA CRC samples harboring different ***KRAS*** mutations. The representations of DEGs from our transcriptome datasets into TCGA transcriptome datasets of colon and rectum samples harboring KRAS G12/C/D/V/13D mutation in comparisons to samples with no mutations in this study. Here, TCGA log_2_FC, the log2FC from TCGA data; DiffExpr log_2_FC, log_2_FC from our data and Directionality, directionality of the expression (positive, if log_2_FC values in both TCGA and our dataset are positive; negative, if log_2_FC values in both TCGA and our dataset are negative and opposing, if log2FC values in both TCGA and our dataset are positive and negative or vice versa). **Supplementary Table 5. **Differentially Expressed Proteins (DEPs) in ***KRAS*** and ***BRAF*** isogenic cell models. Differentially expressed proteins with ㅣlog_2_ FCㅣ ≥ 1 and adjusted *P* value ≤ 0,05 from comparisons of *KRAS* G12C/D/V and G13D to wildtype (3 A, C, E, G) or *BRAF* V600E (3B, D, F, H) and HCT116 BRAF V600E to KRAS G13D. log_2_FC, log_2_-transformed fold change of the expression between contrast groups; AveExpr, Average protein expression; t, moderated t-statistics; P.Value, p-value; Adj.P.val, Benjamini-Hochberg adjusted p-value; B, log-odds of the protein being differentially expressed. **Supplementary Table 6. **Pathway analysis of differentially expressed genes (DEGs) in ***KRAS*** mutant RKO cells. Ingenuity Pathway Analysis (IPA) of differentially expressed genes with ㅣlog_2_ FCㅣ > 1 and FDR ≤ 0.05 from *KRAS* G13D, G12C/D/V knock-ins compared to wild-type and *BRAF* V600E (5 A-D). -log(p-value), -log_10_ of the Benjamini-Hochberg corrected p-value obtained from the Fisher’s Exact test; zScore, A score indicating predicted activation (z-score > 2) or inactivation (z-score < -2) of a pathway in question ; Ratio, no. of differentially expressed genes in a given pathway divided by the total number of genes that make up that pathway and that are in the reference gene set; P-Value, reverse log of the -log_10_ p-values; geneNames, HGNC symbols of the differentially expressed genes associated with the pathway in question; Adj. p-value, p-values adjusted for multiple testing using the Benjamini-Hochberg procedure and cut-off < 0,05. **Supplementary Table 7. **Pathway analysis of differentially expressed proteins (DEPs) in KRAS mutant RKO cells. Ingenuity Pathway Analysis (IPA) of differentially expressed genes with ㅣlog_2_ FCㅣ > 1 and FDR ≤ 0.05 from comparisons of KRAS G12D and G13D to wild-type and BRAF V600E cells (6 A-B). -log(p-value), -log_10_ of the Benjamini-Hochberg corrected p-value obtained from the Fisher’s Exact test; zScore, A score indicating predicted activation (z-score > 2) or inactivation (z-score < -2) of a pathway in question ; Ratio, no. of differentially expressed genes in a given pathway divided by the total number of genes that make up that pathway and that are in the reference gene set; P-Value, reverse log of the -log_10_ p-values; geneNames, HGNC symbols of the differentially expressed genes associated with the pathway in question; Adj. p-value, p-values adjusted for multiple testing using the Benjamini-Hochberg procedure and cut-off < 0,05. **Supplementary Table 8. **Immune related DEGs in KRAS mutant RKO cells. The status of DEGs related to IFN-alpha pathway (Supplementary Tables 2, Additional File 1; Liao et al., 2019) (8 A), IFN-Gamma pathway (Supplementary Tables 2, Additional File 2; Liao et al., 2019) (8B) and immune genes whose genomic deletions were mutually exclusive to KRAS mutations (Supplementary Tables 3, Additional File 2; Liao et al., 2019) (8 C) in our transcriptome dataset. DiffExpr Log_2_FC, -log_2_FC from our transcriptome data. **Supplementary Table 9. **Enrichment of metabolic pathways in KRAS mutant RKO cells. LC-MS metabolomics data were acquired in both positive and negative mode from KRAS G12C/D/V/G13D knock-ins compared to BRAF V600E or wild-type (9 A-H) and HCT116 BRAF V600E to KRAS G13D as well as KRAS WT (9I-J). The Mummichog python package was used to perform pathway analysis on each dataset and the positive mode and negative mode results were combined using Fisher’s method to produce a final p-value (a p-value threshold of 0.05 was applied). **Supplementary Table 10. **Enrichment of Differentially expressed Genes (DEGs) for altered metabolic pathways in ***KRAS*** and ***BRAF*** isogenic cell models. Differentially Expressed Genes (DEGs) for significantly altered metabolic pathways extracted from KEGG pathway database were analyzed on transcriptome dataset (Supplementary table 2 A-J, Additional File 2) for KRAS G12C/D/V/G13D knock-ins compared to BRAF V600E or wild-type (10 A-H) and HCT116 BRAF V600E to KRAS G13D as well as KRAS WT (10I-J) with log2 fold change ≥ 1 and FDR ≤ 0,05. FDR, False Discovery Rate; logFC, log_2_-transformed fold change of expression.

## Data Availability

All the data will be available after publication. No human patient material was used in this study.

## Abbreviations

CRC: Colorectal Cancer
TCGA: The Cancer Genome Atlas
HA1 and 2: Homology Arm 1 and 2
DEGs: Differentially Expressed Genes
DEPs: Differentially Expressed Proteins
IPA: Ingenuity Pathway Assay
TMT: Tandem Mass Tag

## References

[1] Rajagopalan H, Bardelli A, Lengauer C, Kinzler KW, Vogelstein B, Velculescu VE (2002). Tumorigenesis: RAF/RAS oncogenes and mismatch-repair status. Nature.

[2] Morkel M, Riemer P, Bläker H, Sers C (2015). Similar but different: distinct roles for KRAS and BRAF oncogenes in colorectal cancer development and therapy resistance. Oncotarget.

[3] De Roock W, Jonker DJ, Di Nicolantonio F, Sartore-Bianchi A, Tu D, Siena S, Lamba S, Arena S, Frattini M, Piessevaux H (2010). Association of KRAS p.G13D mutation with outcome in patients with chemotherapy-refractory metastatic colorectal cancer treated with cetuximab. JAMA.

[4] Gaglio D, Metallo CM, Gameiro PA, Hiller K, Danna LS, Balestrieri C, Alberghina L, Stephanopoulos G, Chiaradonna F (2011). Oncogenic K-Ras decouples glucose and glutamine metabolism to support cancer cell growth. Mol Syst Biol.

[5] Yun J, Rago C, Cheong I, Pagliarini R, Angenendt P, Rajagopalan H, Schmidt K, Willson JK, Markowitz S, Zhou S (2009). Glucose deprivation contributes to the development of KRAS pathway mutations in tumor cells. Science.

[6] Vartanian S, Bentley C, Brauer MJ, Li L, Shirasawa S, Sasazuki T, Kim JS, Haverty P, Stawiski E, Modrusan Z (2013). Identification of mutant K-Ras-dependent phenotypes using a panel of isogenic cell lines. J Biol Chem.

[7] Hammond DE, Mageean CJ, Rusilowicz EV, Wickenden JA, Clague MJ, Prior IA (2015). Differential reprogramming of isogenic colorectal cancer cells by distinct activating KRAS mutations. J Proteome Res.

[8] Brunelli L, Caiola E, Marabese M, Broggini M, Pastorelli R (2014). Capturing the metabolomic diversity of KRAS mutants in non-small-cell lung cancer cells. Oncotarget.

[9] Preto A, Figueiredo J, Velho S, Ribeiro AS, Soares P, Oliveira C, Seruca R (2008). BRAF provides proliferation and survival signals in MSI colorectal carcinoma cells displaying BRAF(V600E) but not KRAS mutations. J Pathol.

[10] Stoimenov I, Ali MA, Pandzic T, Sjöblom T (2015). Computational and molecular tools for scalable rAAV-mediated genome editing. Nucleic Acids Res.

[11] Krueger F. Trim galore. A wrapper tool around Cutadapt and FastQC to consistently apply quality and adapter trimming to FastQ files 2015, 516:517.

[12] Martin M: Cutadapt removes adapter sequences from high-throughput sequencing reads. 2011 2011, 17:3.

[13] Andrews Sea: FastQC. 2015.

[14] Dobin A, Davis CA, Schlesinger F, Drenkow J, Zaleski C, Jha S, Batut P, Chaisson M, Gingeras TR (2013). STAR: ultrafast universal RNA-seq aligner. Bioinformatics.

[15] Leng N, Kendziorski C: EBSeq: An R package for gene and isoform differential expression analysis of RNA-seq data. R package versions 1.22.1. In.; 2019.

[16] Tang Y, Horikoshi M, Li W. ggfortify: Unified Interface to Visualize Statistical Results of Popular R Packages. The R Journal. January 2016;8(2):478–89.

[17] Kolde R. Pheatmap: pretty heatmaps. R package version 2012, 1.

[18] Chen H, Boutros PC (2011). VennDiagram: a package for the generation of highly-customizable Venn and Euler diagrams in R. BMC Bioinformatics.

[19] Ritchie ME, Phipson B, Wu D, Hu Y, Law CW, Shi W, Smyth GK (2015). limma powers differential expression analyses for RNA-sequencing and microarray studies. Nucleic Acids Res.

[20] Benjamini Y, Hochberg Y. Controlling the False Discovery Rate: A Practical and Powerful Approach to Multiple Testing.

[21] Krämer A, Green J, Pollard J, Tugendreich S (2014). Causal analysis approaches in Ingenuity Pathway Analysis. Bioinformatics.

[22] Brattain MG, Fine WD, Khaled FM, Thompson J, Brattain DE (1981). Heterogeneity of malignant cells from a human colonic carcinoma. Cancer Res..

[23] Brattain MG, Levine AE, Chakrabarty S, Yeoman LC, Willson JK, Long B (1984). Heterogeneity of human colon carcinoma. Cancer Metastasis Rev..

[24] Shirasawa S, Furuse M, Yokoyama N, Sasazuki T (1993). Altered growth of human colon cancer cell lines disrupted at activated Ki-ras. Science.

[25] Smith CA, Want EJ, O’Maille G, Abagyan R, Siuzdak G (2006). XCMS: processing mass spectrometry data for metabolite profiling using nonlinear peak alignment, matching, and identification. Anal Chem.

[26] Li S, Park Y, Duraisingham S, Strobel FH, Khan N, Soltow QA, Jones DP, Pulendran B (2013). Predicting network activity from high throughput metabolomics. PLoS Comput Biol.

[27] Dexter DL, Barbosa JA, Calabresi P (1979). N,N-dimethylformamide-induced alteration of cell culture characteristics and loss of tumorigenicity in cultured human colon carcinoma cells. Cancer Res.

[28] Brattain MG, Brattain DE, Sarrif AM, McRae LJ, Fine WD, Hawkins JG (1982). Enhancement of growth of human colon tumor cell lines by feeder layers of murine fibroblasts. J Natl Cancer Inst.

[29] Rago C, Vogelstein B, Bunz F (2007). Genetic knockouts and knockins in human somatic cells. Nat Protoc.

[30] Ikediobi O, Davies H, Bignell G, Edkins S, Stevens C, O’Meara S, Santarius T, Avis T, Barthorpe S, Brackenbury L (2006). Mutation analysis of 24 known cancer genes in the NCI-60 cell line set. Mol Cancer Ther.

[31] Tsunoda T, Takashima Y, Fujimoto T, Koyanagi M, Yoshida Y, Doi K, Tanaka Y, Kuroki M, Sasazuki T, Shirasawa S (2010). Three-dimensionally specific inhibition of DNA repair-related genes by activated KRAS in colon crypt model. Neoplasia.

[32] Engeland K (2018). Cell cycle arrest through indirect transcriptional repression by p53: I have a DREAM. Cell Death Differ.

[33] Ashery U, Yizhar O, Rotblat B, Elad-Sfadia G, Barkan B, Haklai R, Kloog Y (2006). Spatiotemporal organization of Ras signaling: rasosomes and the galectin switch. Cell Mol Neurobiol.

[34] Ding B, Lengyel P (2008). p204 protein is a novel modulator of ras activity. J Biol Chem.

[35] Madureira PA, Bharadwaj AG, Bydoun M, Garant K, O’Connell P, Lee P, Waisman DM: Cell surface protease activation during RAS transformation: Critical role of the plasminogen receptor, S100A10. Oncotarget 2016, 7:47720–47737. 10.18632/oncotarget.10279 PMC5216974 27351226

[36] Zhao P, Damerow MS, Stern P, Liu AH, Sweet-Cordero A, Siziopikou K, Neilson JR, Sharp PA, Cheng C (2013). CD44 promotes Kras-dependent lung adenocarcinoma. Oncogene.

[37] Vallejo A, Perurena N, Guruceaga E, Mazur PK, Martinez-Canarias S, Zandueta C, Valencia K, Arricibita A, Gwinn D, Sayles LC (2017). An integrative approach unveils FOSL1 as an oncogene vulnerability in KRAS-driven lung and pancreatic cancer. Nat Commun.

[38] Ritchie C, Mack A, Harper L, Alfadhli A, Stork PJS, Nan X, Barklis E (2017). Analysis of K-Ras Interactions by Biotin Ligase Tagging. Cancer Genomics Proteomics.

[39] Okudela K, Katayama A, Woo T, Mitsui H, Suzuki T, Tateishi Y, Umeda S, Tajiri M, Masuda M, Nagahara N (2014). Proteome analysis for downstream targets of oncogenic KRAS–the potential participation of CLIC4 in carcinogenesis in the lung. PLoS One.

[40] Mlakar V, Berginc G, Volavsek M, Stor Z, Rems M, Glavac D (2009). Presence of activating KRAS mutations correlates significantly with expression of tumour suppressor genes DCN and TPM1 in colorectal cancer. BMC Cancer.

[41] Tantyo NA, Karyadi AS, Rasman SZ, Salim MRG, Devina A, Sumarpo A (2019). The prognostic value of S100A10 expression in cancer. Oncol Lett.

[42] Wei L, Lee D, Law CT, Zhang MS, Shen J, Chin DW, Zhang A, Tsang FH, Wong CL, Ng IO (2019). Genome-wide CRISPR/Cas9 library screening identified PHGDH as a critical driver for Sorafenib resistance in HCC. Nat Commun.

[43] Shankar S, Pitchiaya S, Malik R, Kothari V, Hosono Y, Yocum AK, Gundlapalli H, White Y, Firestone A, Cao X (2016). KRAS Engages AGO2 to Enhance Cellular Transformation. Cell Rep.

[44] Ye X, Chan KC, Waters AM, Bess M, Harned A, Wei BR, Loncarek J, Luke BT, Orsburn BC, Hollinger BD (2016). Comparative proteomics of a model MCF10A-KRasG12V cell line reveals a distinct molecular signature of the KRasG12V cell surface. Oncotarget.

[45] Tsoumas D, Nikou S, Giannopoulou E, Champeris Tsaniras S, Sirinian C, Maroulis I, Taraviras S, Zolota V, Kalofonos HP, Bravou V (2018). ILK Expression in Colorectal Cancer Is Associated with EMT, Cancer Stem Cell Markers and Chemoresistance. Cancer Genomics Proteomics.

[46] Zheng CC, Hu HF, Hong P, Zhang QH, Xu WW, He QY, Li B (2019). Significance of integrin-linked kinase (ILK) in tumorigenesis and its potential implication as a biomarker and therapeutic target for human cancer. Am J Cancer Res.

[47] Lal N, Beggs AD, Willcox BE, Middleton GW (2015). An immunogenomic stratification of colorectal cancer: Implications for development of targeted immunotherapy. Oncoimmunology.

[48] Lal N, White BS, Goussous G, Pickles O, Mason MJ, Beggs AD, Taniere P, Willcox BE, Guinney J, Middleton GW (2018). KRAS Mutation and Consensus Molecular Subtypes 2 and 3 Are Independently Associated with Reduced Immune Infiltration and Reactivity in Colorectal Cancer. Clin Cancer Res.

[49] Liao W, Overman MJ, Boutin AT, Shang X, Zhao D, Dey P, Li J, Wang G, Lan Z, Tang M (2019). KRAS-IRF2 Axis Drives Immune Suppression and Immune Therapy Resistance in Colorectal Cancer. Cancer Cell.

[50] Son J, Lyssiotis CA, Ying H, Wang X, Hua S, Ligorio M, Perera RM, Ferrone CR, Mullarky E, Shyh-Chang N (2013). Glutamine supports pancreatic cancer growth through a KRAS-regulated metabolic pathway. Nature.

[51] Brunelli L, Caiola E, Marabese M, Broggini M, Pastorelli R (2016). Comparative metabolomics profiling of isogenic KRAS wild type and mutant NSCLC cells in vitro and in vivo. Sci Rep.

[52] Longo N, Frigeni M, Pasquali M (2016). Carnitine transport and fatty acid oxidation. Biochim Biophys Acta.

[53] Nigam SK (2018). The SLC22 Transporter Family: A Paradigm for the Impact of Drug Transporters on Metabolic Pathways, Signaling, and Disease. Annu Rev Pharmacol Toxicol.

[54] Nezu J, Tamai I, Oku A, Ohashi R, Yabuuchi H, Hashimoto N, Nikaido H, Sai Y, Koizumi A, Shoji Y (1999). Primary systemic carnitine deficiency is caused by mutations in a gene encoding sodium ion-dependent carnitine transporter. Nat Genet.

[55] Muñoz-Maldonado C, Zimmer Y, Medová M (2019). A Comparative Analysis of Individual RAS Mutations in Cancer Biology. Front Oncol.

[56] Pantsar T (2020). The current understanding of KRAS protein structure and dynamics. Comput Struct Biotechnol J.

[57] Ihle NT, Byers LA, Kim ES, Saintigny P, Lee JJ, Blumenschein GR, Tsao A, Liu S, Larsen JE, Wang J (2012). Effect of KRAS oncogene substitutions on protein behavior: implications for signaling and clinical outcome. J Natl Cancer Inst.

[58] Gao Q, Zhu H, Dong L, Shi W, Chen R, Song Z, Huang C, Li J, Dong X, Zhou Y (2019). Integrated Proteogenomic Characterization of HBV-Related Hepatocellular Carcinoma. Cell.

[59] Mun DG, Bhin J, Kim S, Kim H, Jung JH, Jung Y, Jang YE, Park JM, Lee H, Bae J (2019). Proteogenomic Characterization of Human Early-Onset Gastric Cancer. Cancer Cell.

[60] Zhang B, Wang J, Wang X, Zhu J, Liu Q, Shi Z, Chambers MC, Zimmerman LJ, Shaddox KF, Kim S (2014). Proteogenomic characterization of human colon and rectal cancer. Nature.

[61] Barras D, Missiaglia E, Wirapati P, Sieber OM, Jorissen RN, Love C, Molloy PL, Jones IT, McLaughlin S, Gibbs P (2017). BRAF V600E Mutant Colorectal Cancer Subtypes Based on Gene Expression. Clin Cancer Res.

[62] Wiesweg M, Kasper S, Worm K, Herold T, Reis H, Sara L, Metzenmacher M, Abendroth A, Darwiche K, Aigner C (2019). Impact of RAS mutation subtype on clinical outcome-a cross-entity comparison of patients with advanced non-small cell lung cancer and colorectal cancer. Oncogene.

[63] Alajez NM (2016). Large-Scale Analysis of Gene Expression Data Reveals a Novel Gene Expression Signature Associated with Colorectal Cancer Distant Recurrence. PLoS One.

[64] Li C, Chen Q, Zhou Y, Niu Y, Wang X, Li X, Zheng H, Wei T, Zhao L, Gao H (2020). S100A2 promotes glycolysis and proliferation via GLUT1 regulation in colorectal cancer. FASEB J.

[65] Masuda T, Ishikawa T, Mogushi K, Okazaki S, Ishiguro M, Iida S, Mizushima H, Tanaka H, Uetake H, Sugihara K (2016). Overexpression of the S100A2 protein as a prognostic marker for patients with stage II and III colorectal cancer. Int J Oncol.

[66] Shang J, Zhang Z, Song W, Zhou B, Zhang Y, Li G, Qiu S (2013). S100A10 as a novel biomarker in colorectal cancer. Tumour Biol.

[67] Wang C, Zhang C, Li X, Shen J, Xu Y, Shi H, Mu X, Pan J, Zhao T, Li M (2019). CPT1A-mediated succinylation of S100A10 increases human gastric cancer invasion. J Cell Mol Med.

[68] Tang W, Zhou M, Dorsey TH, Prieto DA, Wang XW, Ruppin E, Veenstra TD, Ambs S (2018). Integrated proteotranscriptomics of breast cancer reveals globally increased protein-mRNA concordance associated with subtypes and survival. Genome Med.

[69] Tian S, Simon I, Moreno V, Roepman P, Tabernero J, Snel M, van’t Veer L, Salazar R, Bernards R, Capella G (2013). A combined oncogenic pathway signature of BRAF, KRAS and PI3KCA mutation improves colorectal cancer classification and cetuximab treatment prediction. Gut.

[70] Győrffy B, Stelniec-Klotz I, Sigler C, Kasack K, Redmer T, Qian Y, Schäfer R (2015). Effects of RAL signal transduction in KRAS- and BRAF-mutated cells and prognostic potential of the RAL signature in colorectal cancer. Oncotarget.

[71] Yan Z, Yin H, Wang R, Wu D, Sun W, Liu B, Su Q (2014). Overexpression of integrin-linked kinase (ILK) promotes migration and invasion of colorectal cancer cells by inducing epithelial-mesenchymal transition via NF-κB signaling. Acta Histochem.

[72] Chu PC, Yang MC, Kulp SK, Salunke SB, Himmel LE, Fang CS, Jadhav AM, Shan YS, Lee CT, Lai MD (2016). Regulation of oncogenic KRAS signaling via a novel KRAS-integrin-linked kinase-hnRNPA1 regulatory loop in human pancreatic cancer cells. Oncogene.

[73] Nikou S, Arbi M, Dimitrakopoulos FD, Sirinian C, Chadla P, Pappa I, Ntaliarda G, Stathopoulos GT, Papadaki H, Zolota V (2020). Integrin-linked kinase (ILK) regulates KRAS, IPP complex and Ras suppressor-1 (RSU1) promoting lung adenocarcinoma progression and poor survival. J Mol Histol.

[74] Thorsson V, Gibbs DL, Brown SD, Wolf D, Bortone DS, Ou Yang TH, Porta-Pardo E, Gao GF, Plaisier CL, Eddy JA (2019). The Immune Landscape of Cancer. Immunity.

[75] Varshavi D, McCarthy N, Veselkov K, Keun HC, Everett JR (2020). Metabolic characterization of colorectal cancer cells harbouring different KRAS mutations in codon 12, 13, 61 and 146 using human SW48 isogenic cell lines. Metabolomics.

[76] Oba D, Inoue SI, Miyagawa-Tomita S, Nakashima Y, Niihori T, Yamaguchi S, Matsubara Y, Aoki Y (2018). Mice with an Oncogenic HRAS Mutation are Resistant to High-Fat Diet-Induced Obesity and Exhibit Impaired Hepatic Energy Homeostasis. EBioMedicine.

[77] Sung GJ, Choi HK, Kwak S, Song JH, Ko H, Yoon HG, Kang HB, Choi KC (2016). Targeting CPT1A enhances metabolic therapy in human melanoma cells with the BRAF V600E mutation. Int J Biochem Cell Biol.

[78] Quijano C, Cao L, Fergusson MM, Romero H, Liu J, Gutkind S, Rovira II, Mohney RP, Karoly ED, Finkel T (2012). Oncogene-induced senescence results in marked metabolic and bioenergetic alterations. Cell Cycle.

[79] Ratheiser K, Schneeweiss B, Waldhäusl W, Fasching P, Korn A, Nowotny P, Rohac M, Wolf HP (1991). Inhibition by etomoxir of carnitine palmitoyltransferase I reduces hepatic glucose production and plasma lipids in non-insulin-dependent diabetes mellitus. Metabolism.

[80] Beadle RM, Frenneaux M (2010). Modification of myocardial substrate utilisation: a new therapeutic paradigm in cardiovascular disease. Heart.

[81] Cheng S, Wang G, Wang Y, Cai L, Qian K, Ju L, Liu X, Xiao Y, Wang X (2019). Fatty acid oxidation inhibitor etomoxir suppresses tumor progression and induces cell cycle arrest via PPARγ-mediated pathway in bladder cancer. Clin Sci (Lond).

[82] O’Connor RS, Guo L, Ghassemi S, Snyder NW, Worth AJ, Weng L, Kam Y, Philipson B, Trefely S, Nunez-Cruz S (2018). The CPT1a inhibitor, etomoxir induces severe oxidative stress at commonly used concentrations. Sci Rep.

