# Supplementary material for: Common and mutation specific phenotypes of KRAS and BRAF mutations in colorectal cancer cells revealed by integrative -omics analysis
Source: J Exp Clin Cancer Res. 2021 Jul 7;40:225. doi: 10.1186/s13046-021-02025-2 (PMC8265010; doi:10.1186/s13046-021-02025-2)
Supplement: Supplementary file 1 — Additional file 1:Supplementary Figure S1. Knock-in of KRAS G12C/D/V/13D in human RKO colorectal cancer cells by homologous recombination. A An AAV gene targeting construct was designed to introduce the KRAS G12C/D/V/13D allele in the second exon of KRAS in RKO BRAF WT cells. Numbers indicate primers used for homology arm amplification (7–10) (Supplementary Tables 1, Additional File 2), PCR screening for construct integration (11 and 12) and Cre-mediated removal of the resistance marker (13 and 14) (Supplementary Tables 1, Additional File 2). B PCR detection of targeted KRAS alleles in RKO BRAF WT human colorectal cancer cells in three independent clones. The PCR products of 1181 and 1050 bp represents wild type and targeted alleles, respectively. Supplementary Fig. 2. Detection of knock-in alleles by Sanger sequencing. Sanger sequencing (Supplementary Table 1) was performed using cDNAs from two different clones of (A) RKO KRAS G12C/D/V/13D knock-ins as well as single clone of RKO BRAF WT isogenic control, and from single clone (B) HCT116 BRAF V600E knock-in and HCT116 KRAS WT isogenic control. Supplementary Fig. 3. Validation of expression KRAS knock-in alleles by transcriptome sequencing. Illumina-based transcriptome sequencing (~ 25 M reads/sample) using total RNAs from two different clones of (A) RKO KRAS WT control, (B-E) G12C/D/V and G13D knock-ins and from single clone (F) HCT116 KRAS WT isogenic control as well as (G) HCT116 BRAF V600E knock-in. Presence of knock-in alleles in the transcriptome datasets were visualized in IGV. Supplementary Fig. 4. Differentially Expressed Genes (DEGs) from transcriptomes of KRAS mutant cells. Heatmap representations of the top 100 normalized DEGs with ㅣlog2 FCㅣ > 1 and adjusted P < 0.05 from comparisons of KRAS mutations (A) G12C, (B) G12D, (C) G12V and (D) G13D to wildtype (upper panel) and BRAF V600E (lower panel) RKO cells were represented with heatmaps. Similarly, heatmaps of DEGs from comparisons of (E) HCT116 BRAF V60 [file 13046_2021_2025_MOESM1_ESM.pdf]

Supplementary Information

Common and mutation specific phenotypes of *KRAS* and *BRAF* mutations in colorectal cancer cells revealed by integrative -omics analysis

Snehangshu Kundu[1,*,#], Muhammad Akhtar Ali[1,2,*], Niklas Handin[3], Louis Conway[4], Veronica Rendo[1], Per Artursson[3], Liqun He[1], Daniel Globisch[4] and Tobias Sjöblom[1,#]

[1]Science For Life Laboratory, Department of Immunology, Genetics and Pathology, Uppsala University, SE-751 85 Uppsala, Sweden.

[2]Current affiliation: School of Biological Sciences, University of the Punjab, Lahore.

[3]Department of Pharmacy, Uppsala University SE-751 23 Uppsala, Sweden

[4]Department of Chemistry - BMC, Uppsala University, SE-751 24 Uppsala, Sweden

[*] Equally contributing authors

[#] To whom correspondence should be addressed at snehangshu.kundu@igp.uu.se and tobias.sjoblom@igp.uu.se.

Running title: Integrative analysis of mutant *KRAS* and *BRAF* signaling in colorectal cancers

Key words: Ras pathway, *KRAS*, *BRAF*, colorectal cancer, isogenic cell models, integrative -omics analysis

Additional File 1: Supplementary Figures with legends

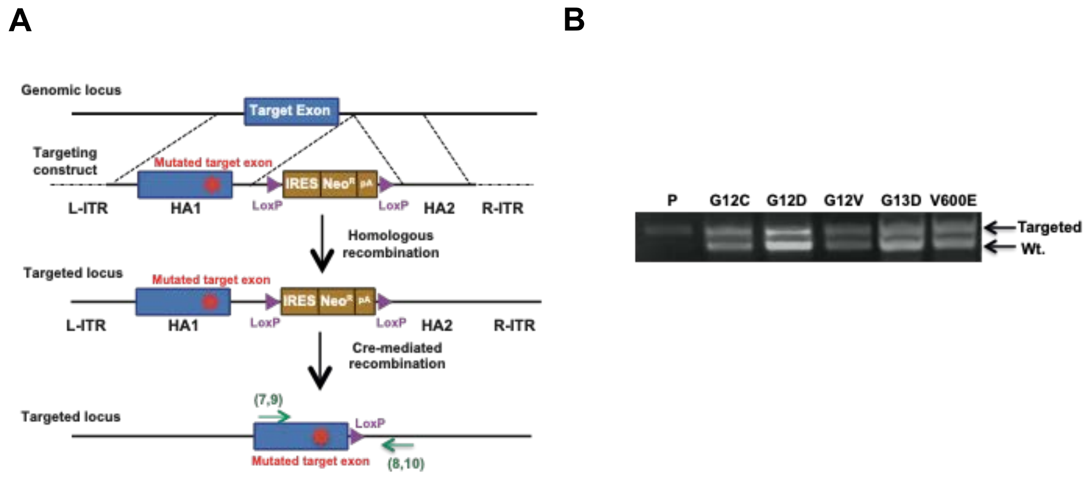

Supplementary figure 1. Knock-in of *KRAS* G12C/D/V/13D in human RKO colorectal cancer cells by homologous recombination. A An AAV gene targeting construct was designed to introduce the *KRAS G12C/D/V/13D* allele in the second exon of *KRAS* in RKO BRAF WT cells. Numbers indicate primers used for homology arm amplification (7-10) (Supplementary table 1), PCR screening for construct integration (11 and 12) and Cre-mediated removal of the resistance marker (13 and 14) (Supplementary table 1). **B** PCR detection of targeted *KRAS* alleles in RKO *BRAF WT* human colorectal cancer cells in three independent clones. The PCR products of 1181 bp and 1050 bp represents wild ty pe and targeted alleles, respectively.

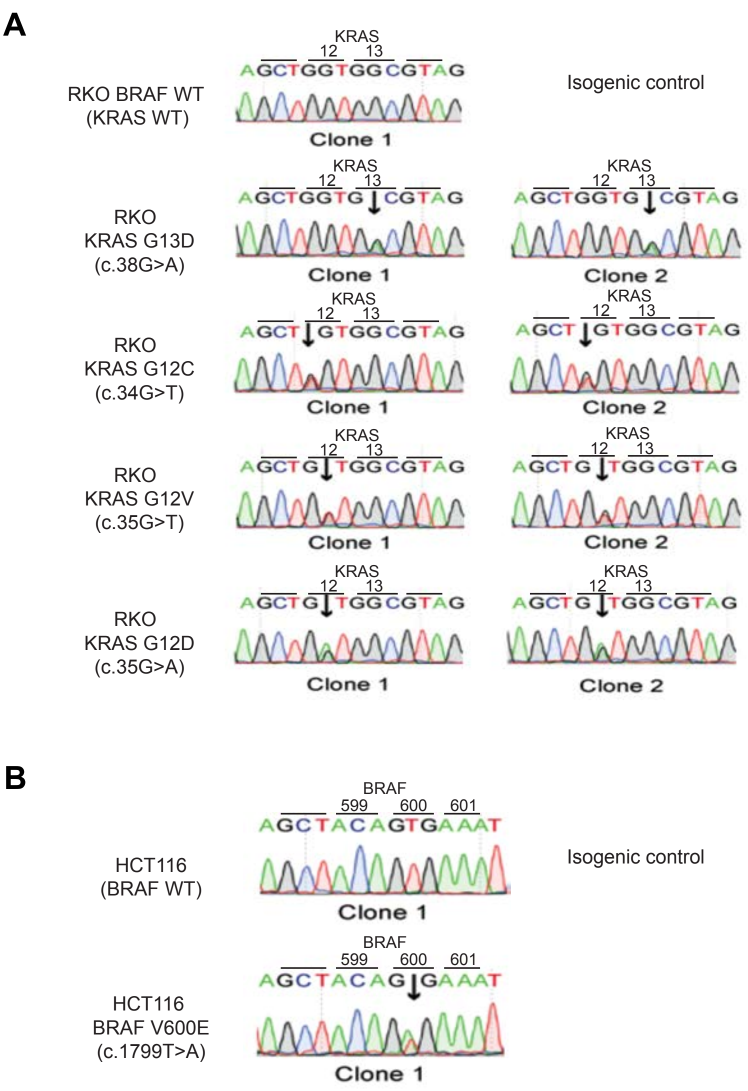

Supplementary figure 2. Detection of knock-in alleles by Sanger sequencing. Sanger sequencing (Supplementary table 1) was performed using cDNAs from two different clones of (**A**) RKO *KRAS* G12C/D/V/13D knock-ins as well as single clone of RKO BRAF WT isogenic control, and from single clone (**B**) HCT116 *BRAF* V600E knock-in and HCT116 KRAS WT isogenic control.

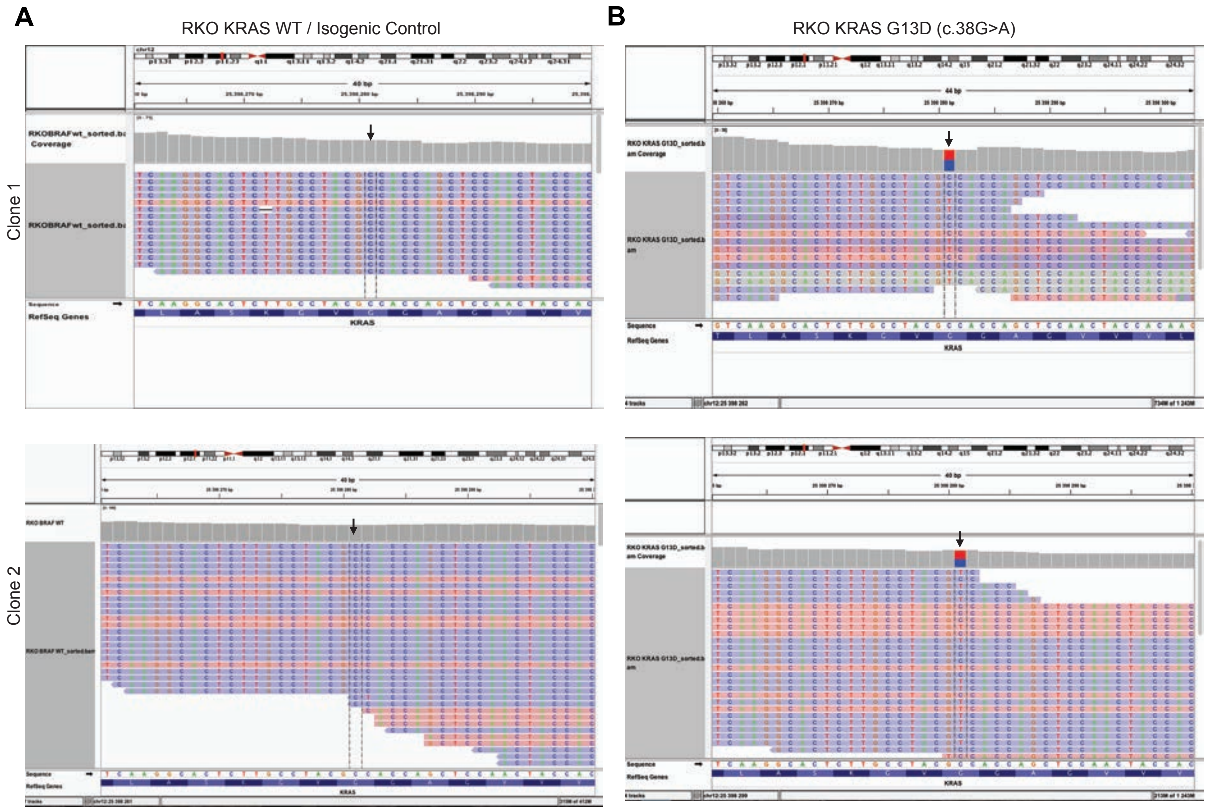
A
RKO KRAS WT / Isogenic Control
B
RKO KRAS G13D (c.38G>A)
Clone 1
Clone 2
KRAS

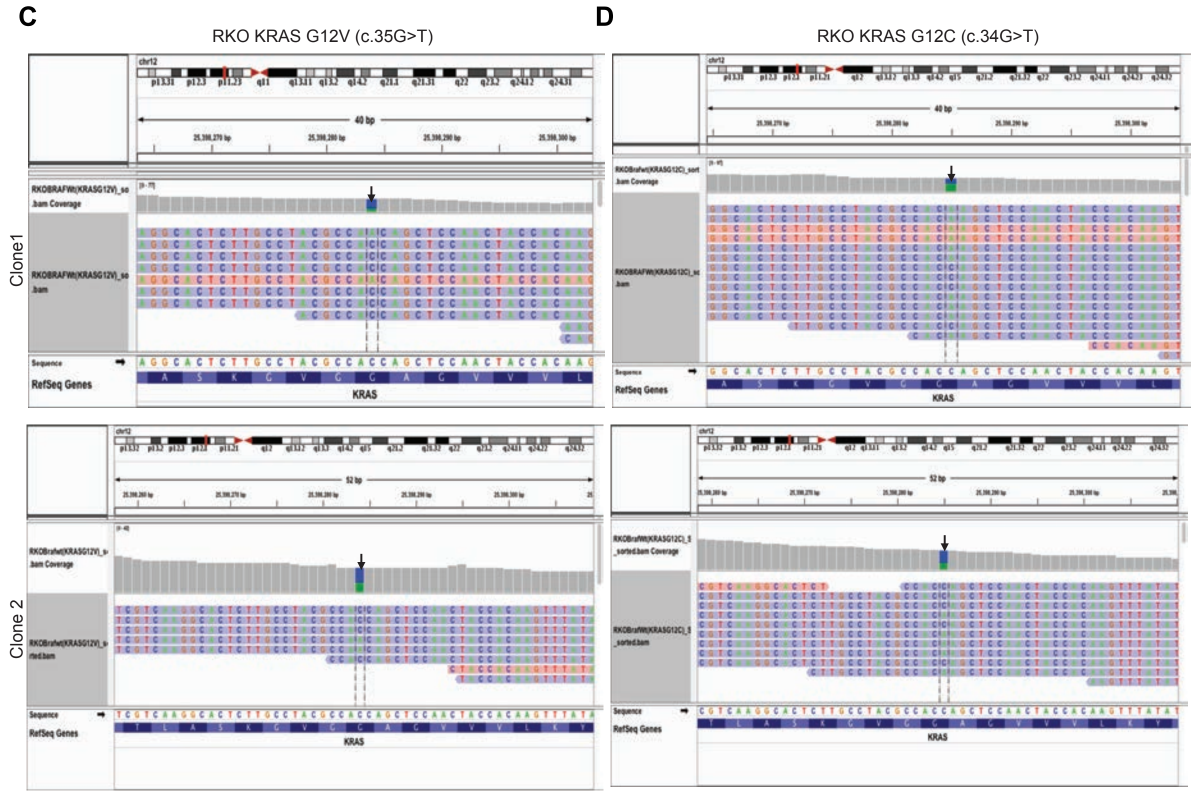
C
RKO KRAS G12V (c.35G>T)
D
RKO KRAS G12C (c.34G>T)
Clone1
Clone 2
RefSeq Genes
KRAS

E RKO KRAS G12D (c.35G>A)

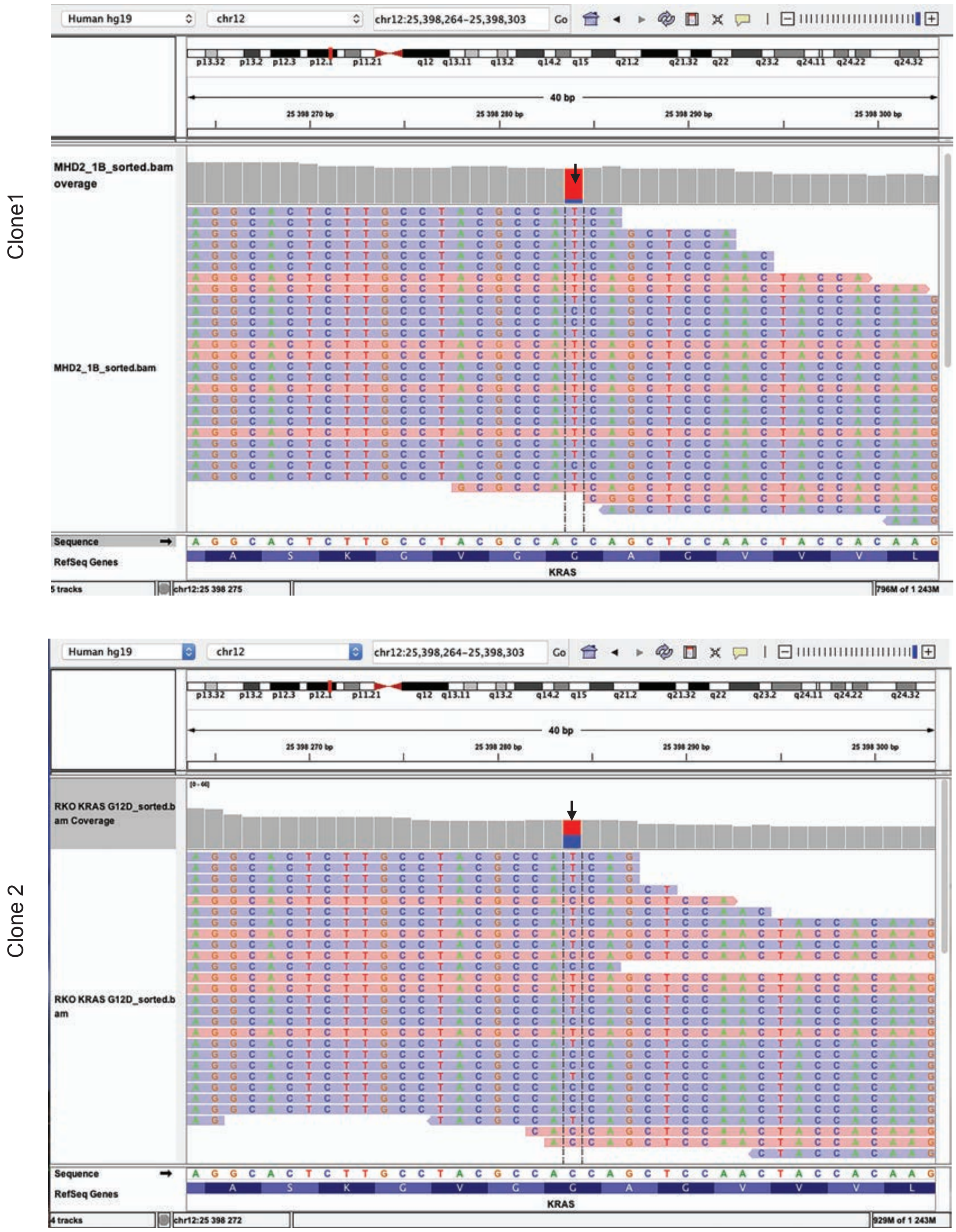

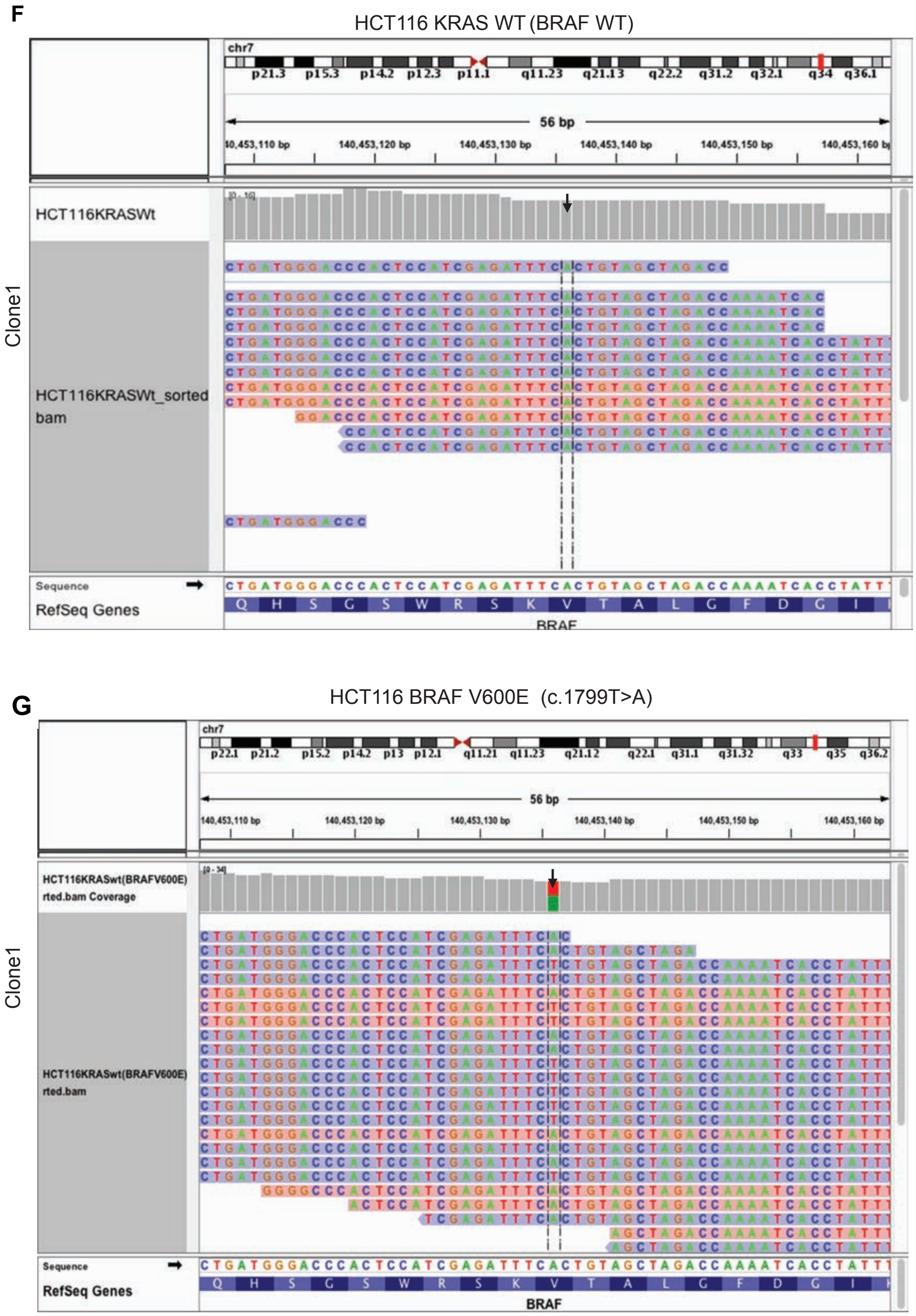

Supplementary figure 3. Validation of expression *KRAS* knock-in alleles by transcriptome sequencing. Illumina-based transcriptome sequencing (~25M reads/sample) using total RNAs from two different clones of (**A**) RKO KRAS WT control, (**B-E**) G12C/D/V and G13D knock-ins and from single clone (**F**) HCT116 KRAS WT isogenic control as well as (**G**) HCT116 *BRAF* V600E knock-in. Presence of knock-in alleles in the transcriptome datasets were visualized in IGV.

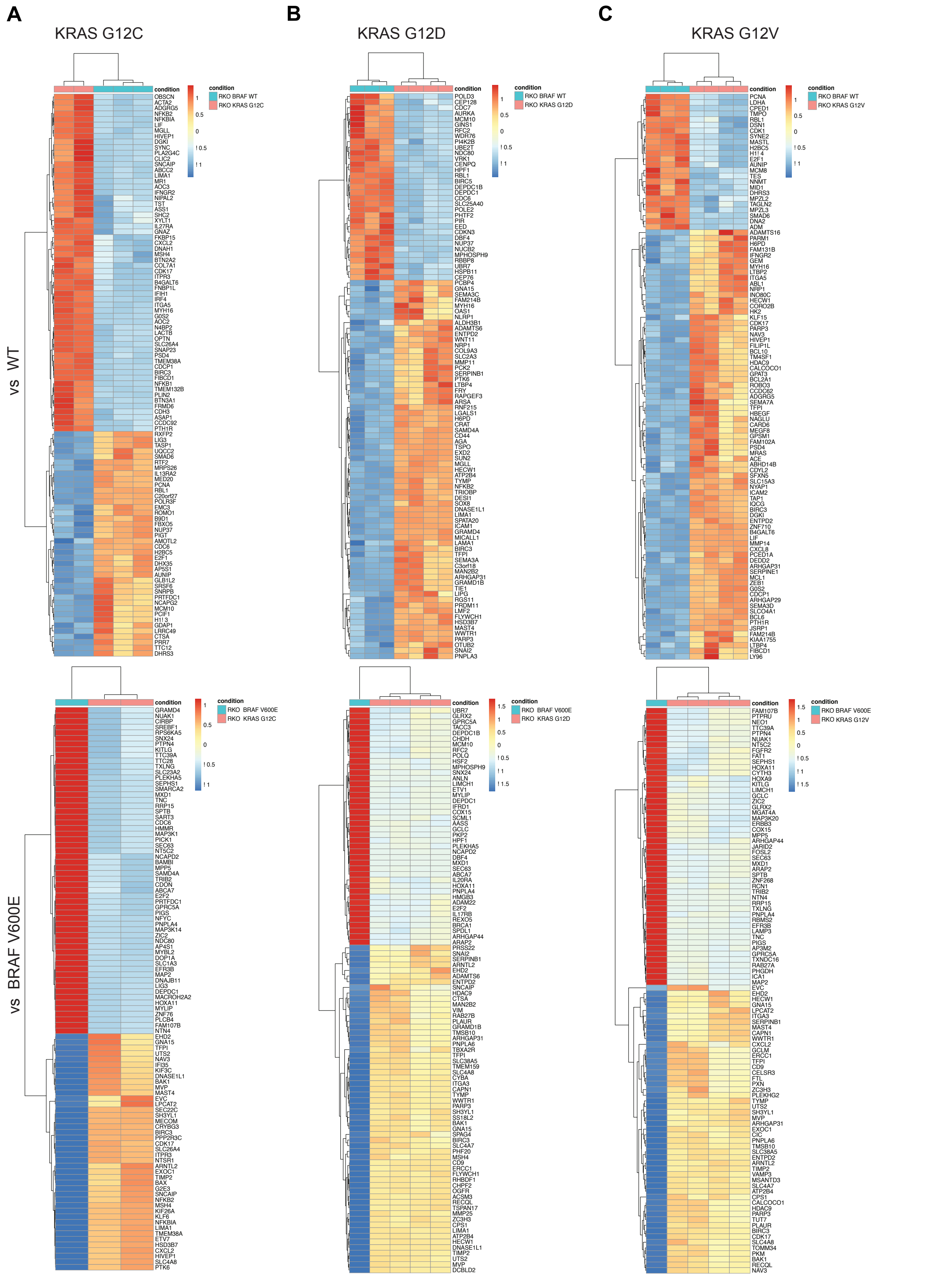
A
KRAS G12C
B
KRAS G12D
C
KRAS G12V
vs WT
vs BRAF V600E
condition
RKO BRAF WT
RKO KRAS G12C
RKO KRAS G12D
RKO KRAS G12V
RKO BRAF V600E

42

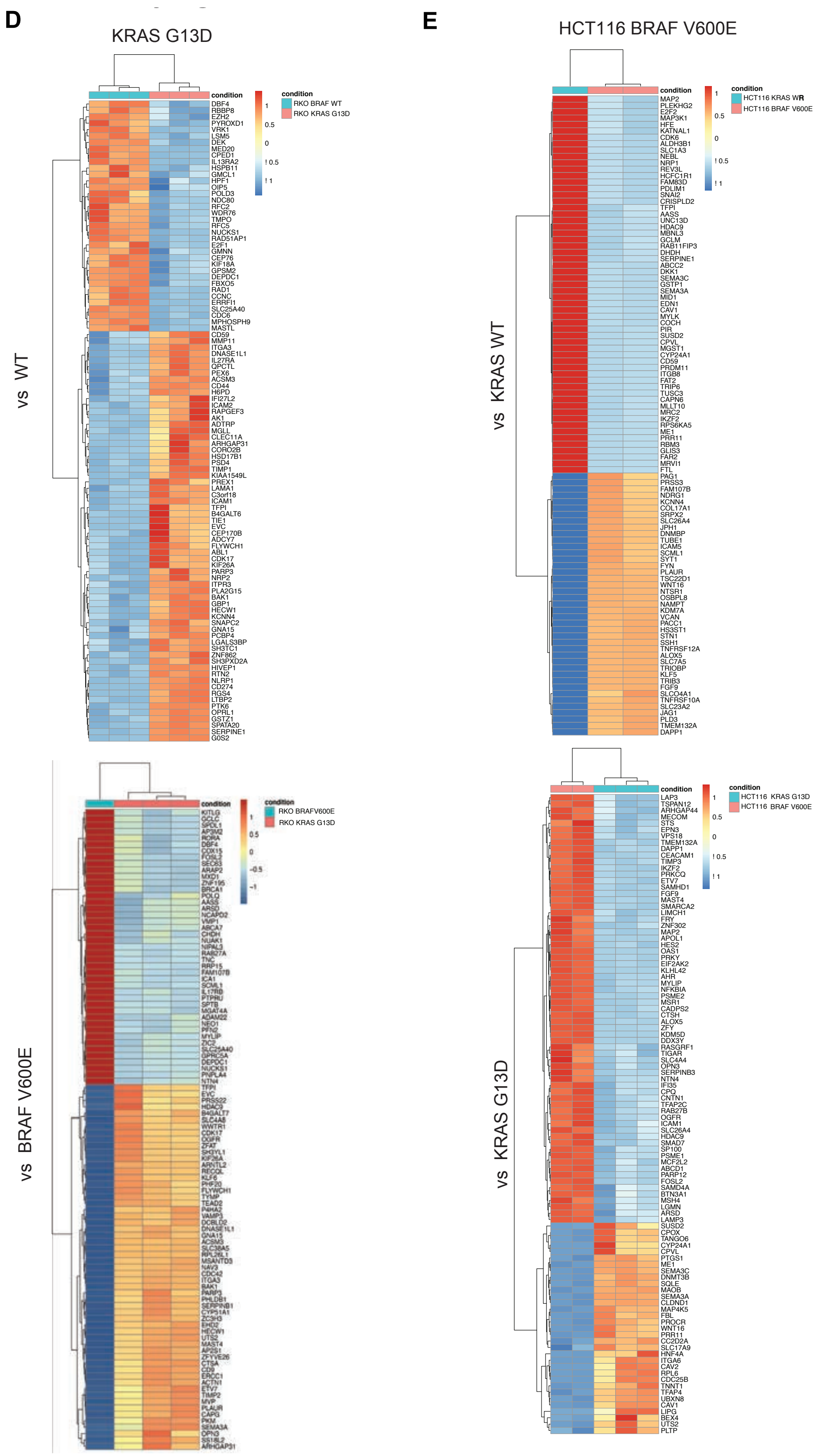

Supplementary figure 4. Differentially Expressed Genes (DEGs) from transcriptomes of *KRAS* mutant cells. Heatmap representations of the top 100 normalized DEGs with $|\log_2 FC| > 1$ and adjusted $P < 0.05$ from comparisons of KRAS mutations (**A**) G12C, (**B**) G12D, (**C**) G12V and (**D**) G13D to wildtype (upper panel) and BRAF V600E (lower panel) RKO cells were represented with heatmaps. Similarly, heatmaps of DEGs from

comparisons of (**E**) HCT116 BRAF V600E vs HCT116 KRAS G12D (upper panel) and HCT116 KRAS WT (lower panel). Transcriptome data was normalized using the EBseq R package (Leng and Kendziorski 2019). Both samples and genes were clustered by Euclidian distances and row-wise scaling was applied to the 100 DEGs with lowest adjusted *p*-values to generate the heatmaps.

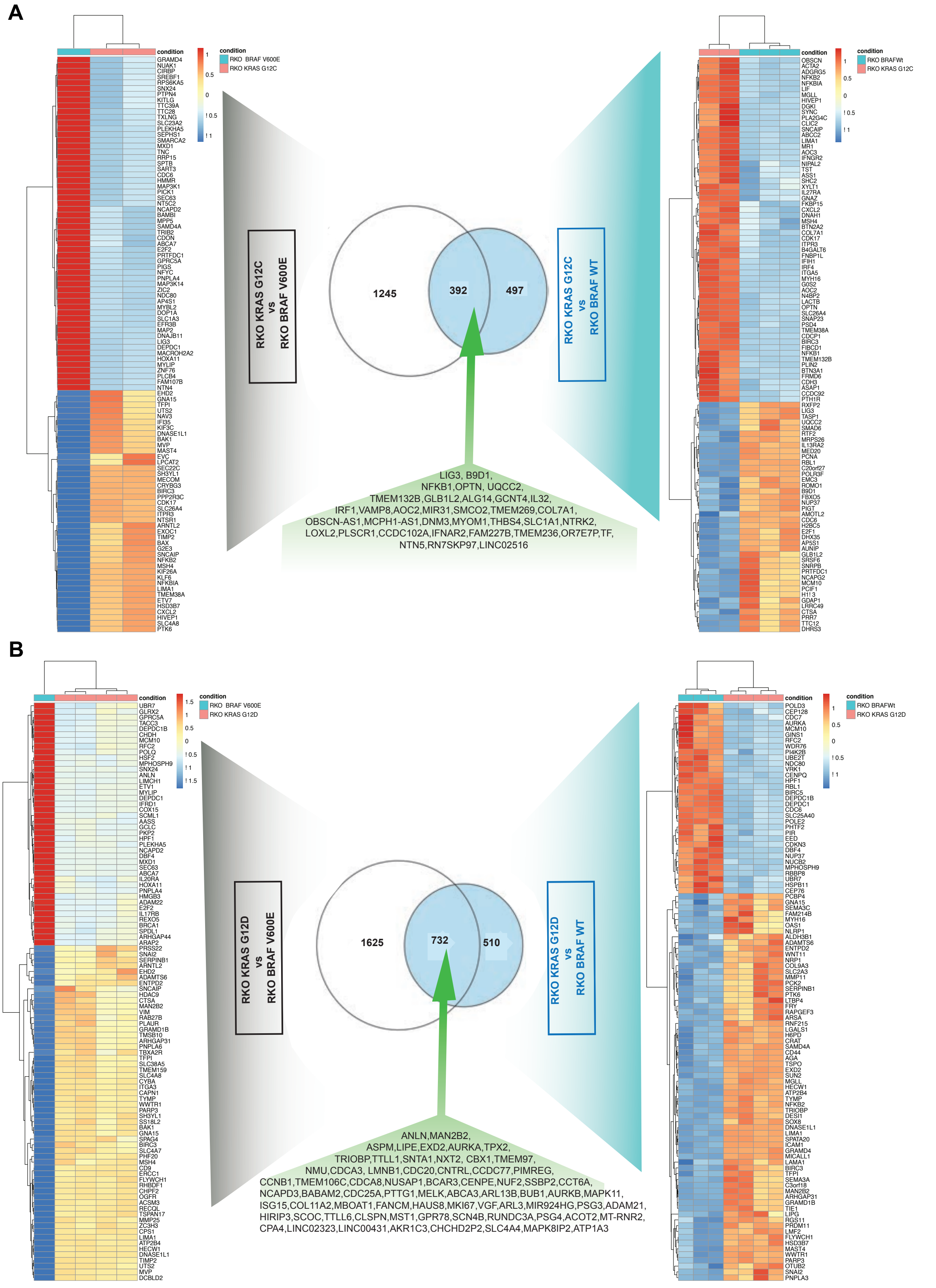

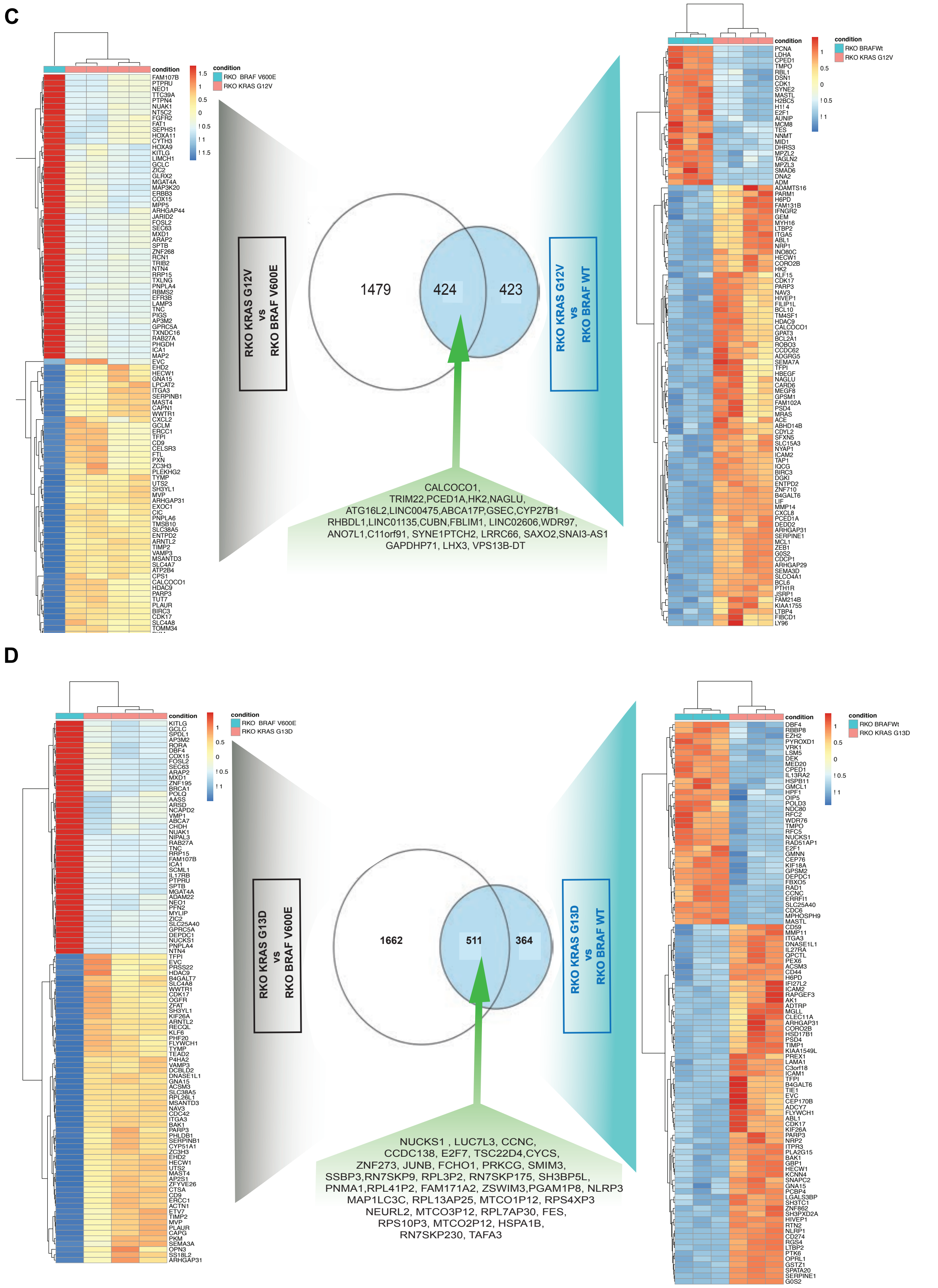
C
RKO KRAS G12V vs RKO BRAF V600E
1479
424
423
RKO KRAS G12V vs RKO BRAF WT
CALCOCO1, TRIM22,PCED1A,HK2,NAGLU, ATG16L2,LINC00475,ABCA17P,GSEC,CYP27B1 RHBDL1,LINC01135,CUBN,FBLIM1, LINC02606,WDR97, ANO7L1,C11orf91, SYNE1PTCH2, LRRC66, SAXO2,SNAI3-AS1 GAPDHP71, LHX3, VPS13B-DT
D
RKO KRAS G13D vs RKO BRAF V600E
1662
511
364
RKO KRAS G13D vs RKO BRAF WT
NUCKS1 , LUC7L3, CCNC, CCDC138, E2F7, TSC22D4,CYCS, ZNF273, JUNB, FCHO1, PRKCG, SMIM3, SSBP3,RN7SKP9, RPL3P2, RN7SKP175, SH3BP5L, PNMA1,RPL41P2, FAM171A2, ZSWIM3,PGAM1P8, NLRP3 MAP1LC3C, RPL13AP25, MTCO1P12, RPS4XP3 NEURL2, MTCO3P12, RPL7AP30, FES, RPS10P3, MTCO2P12, HSPA1B, RN7SKP230, TAFA3

E

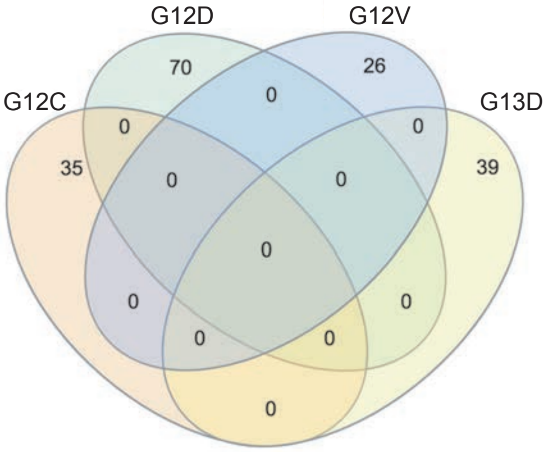

F

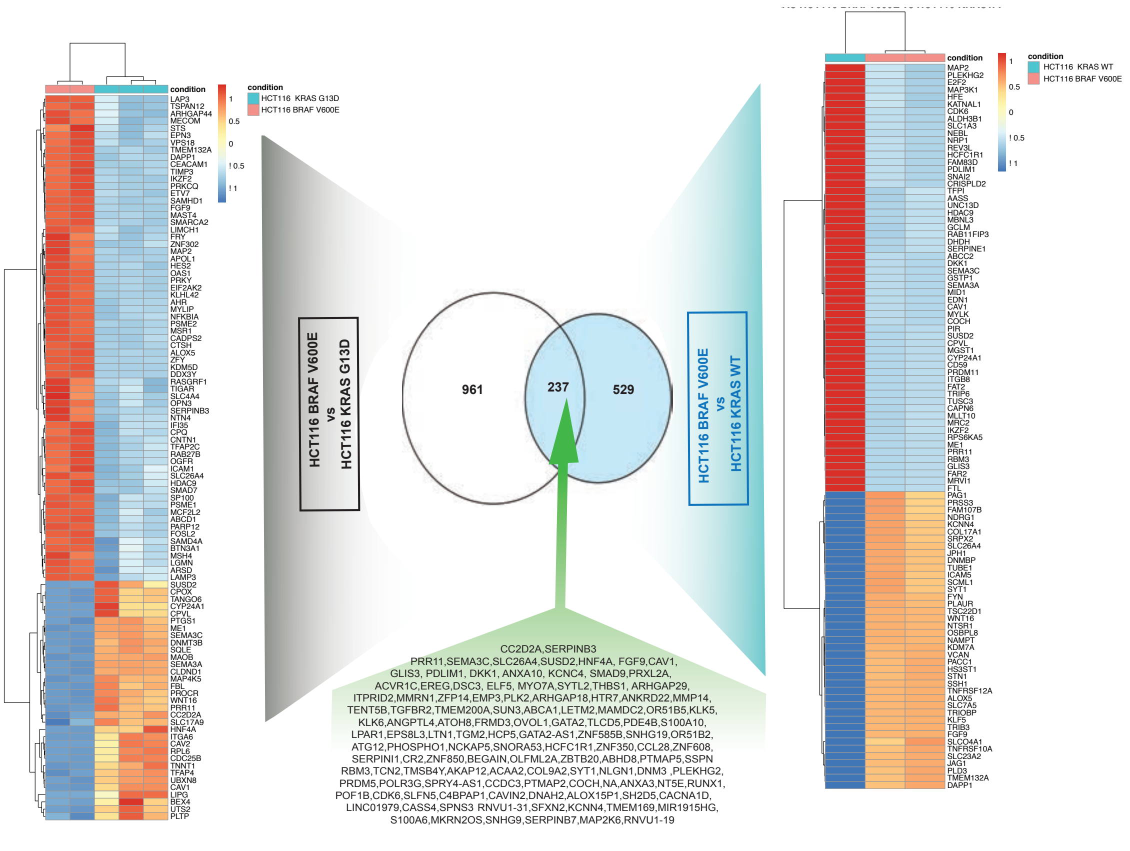

Supplementary figure 5. Mutation specific Differentially Expressed Genes in *KRAS* G12C/D/V/13D and BRAF V600E cells. To identify genes regulated by a specific Ras mutation, the DEGs from comparisons of KRAS mutant cells to wildtype and BRAF V600E cells were intersected and DEGs having the same direction of expression change (i.e. upregulation or downregulation in both wildtype and V600E comparisons) were selected (green field) for (**A**) G12C, (**B**) G12D, (**C**) G12V, (**D**) G13D, respectively. The KRAS mutation specific DEGs

were represented in 4-way Venn diagram (**E**). Similarly, BRAF V600E specific DEGs were identified in HCT116 cells (**F**).

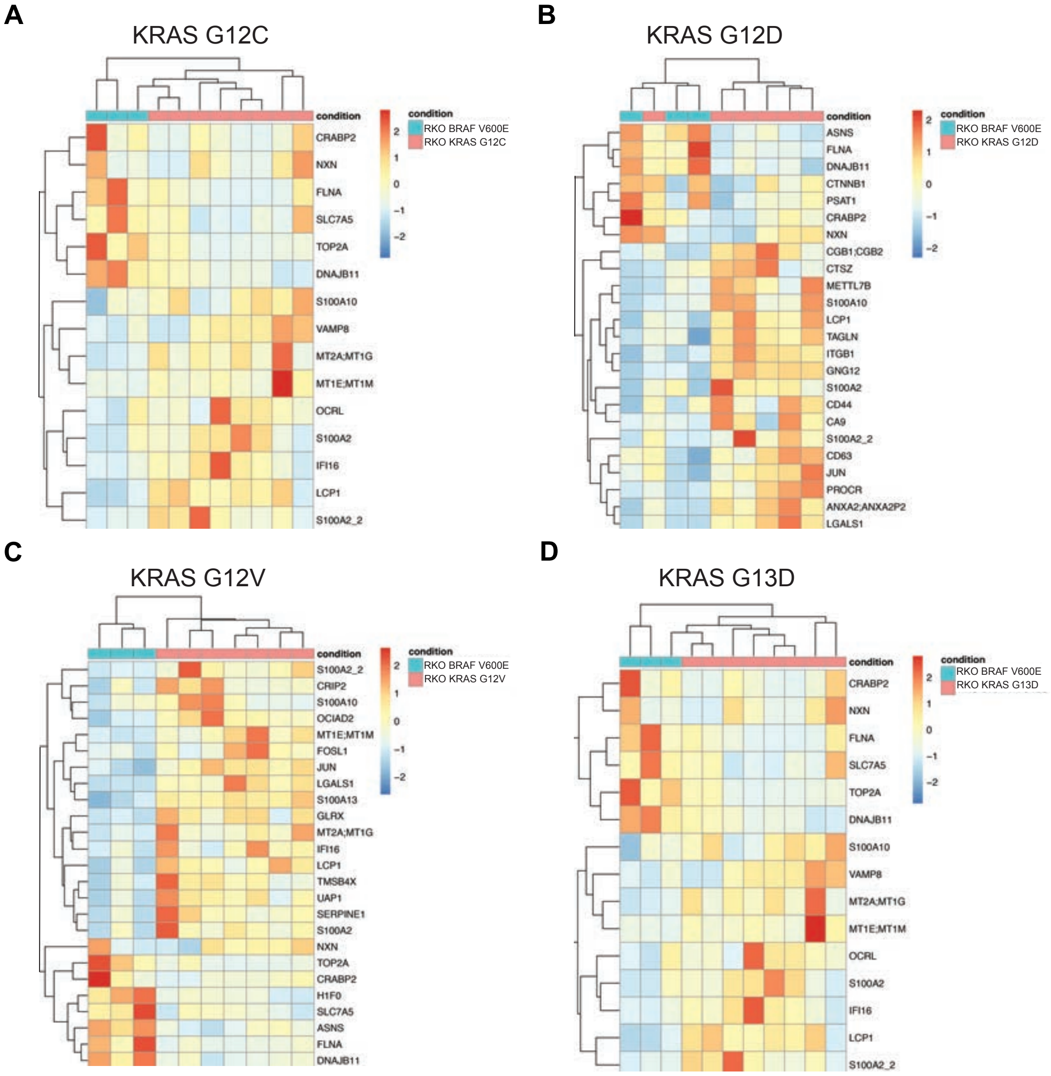

Supplementary figure 6. Differentially Expressed Proteins (DEPs) in *KRAS* versus *BRAF* mutant RKO cells. Top 50 DEPs with $|\log_2 FC| > 1$ and adjusted $P < 0.05$ from the proteome datasets of the comparisons of (**A**) G12C, (**B**) G12D, (**C**) G12V and (**D**) G13D KRAS mutants to BRAF V600E cells were represented with the lowest adjusted *p*-values. Data from LC-MS based proteomic analysis after normalization using quantile normalization and $\log_2$ transformation. Both samples and genes were clustered following Euclidian distances and row-wise scaling was applied to 50 DEPs with lowest adjusted p-values to create the heatmaps.

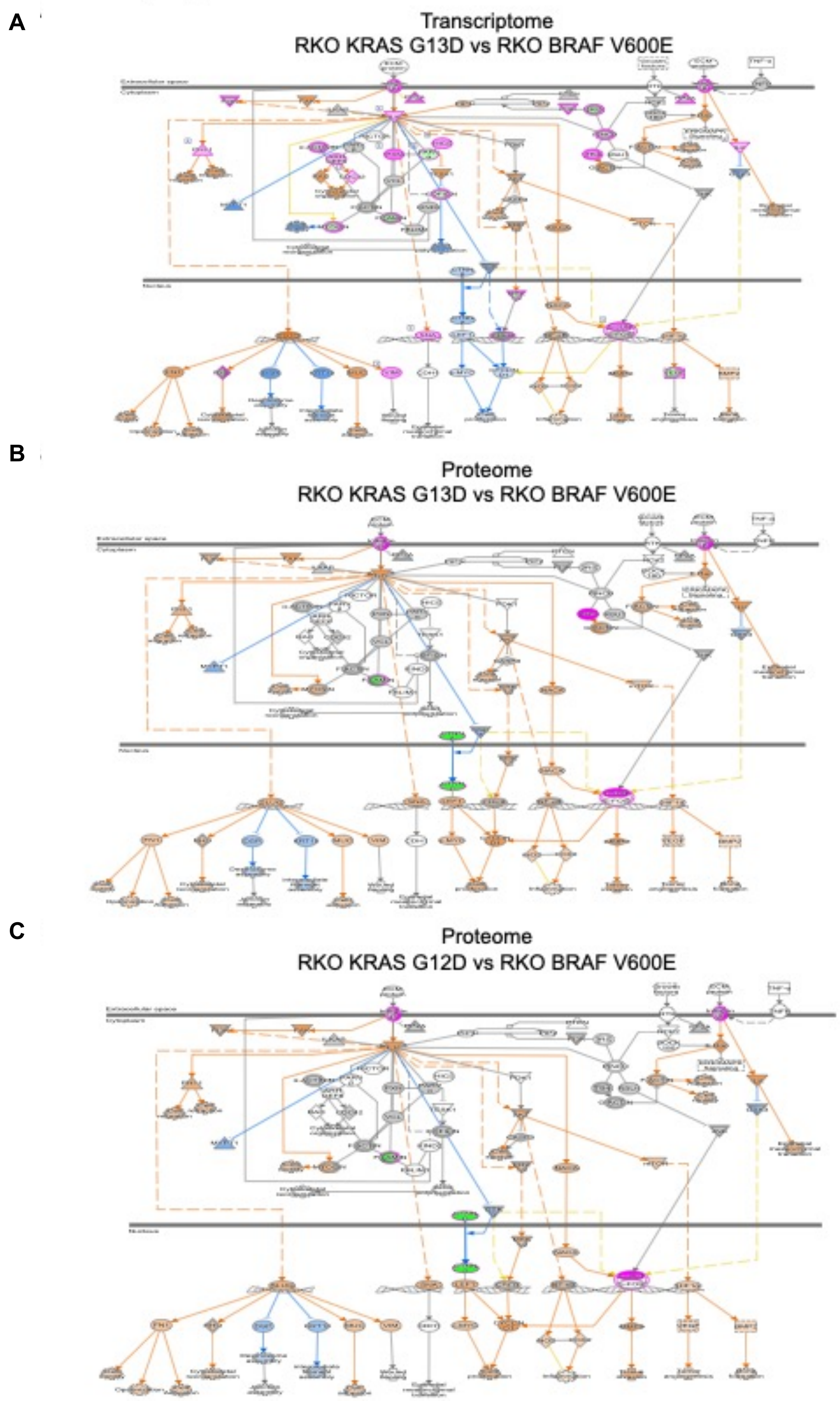

Supplementary figure 7. The ILK signaling pathway is altered at both RNA and protein level by KRAS but not *BRAF* mutation. The ILK pathway was enriched in IPA analysis of DEGs and DEPs fulfilling $|\log_2 \mathrm{FC}| > 1$, adjusted $P < 0.05$ and Z-score ≥ 2 or ≤ -2 in transcriptome data from (**A**) *KRAS* G13D vs *BRAF* V600E and proteomic dataset from (**B**) *KRAS* G12D vs *BRAF* V600E and (**C**) *KRAS* G13D vs *BRAF* V600E comparisons,

respectively. The IPA overlay tool was used for overlaying the expression fold change values of the differential expression analysis followed by the analysis with IPA Molecular Activity Prediction (MAP) tool to predict *in silico* down-stream and up-stream activation or inhibition of molecules and/or processes (cell proliferation, adhesion, tumor angiogenesis and tissue invasion) based on the observed expression changes due to KRAS G12D/13D mutations. Pink, up-regulated genes; green, down-regulated genes.

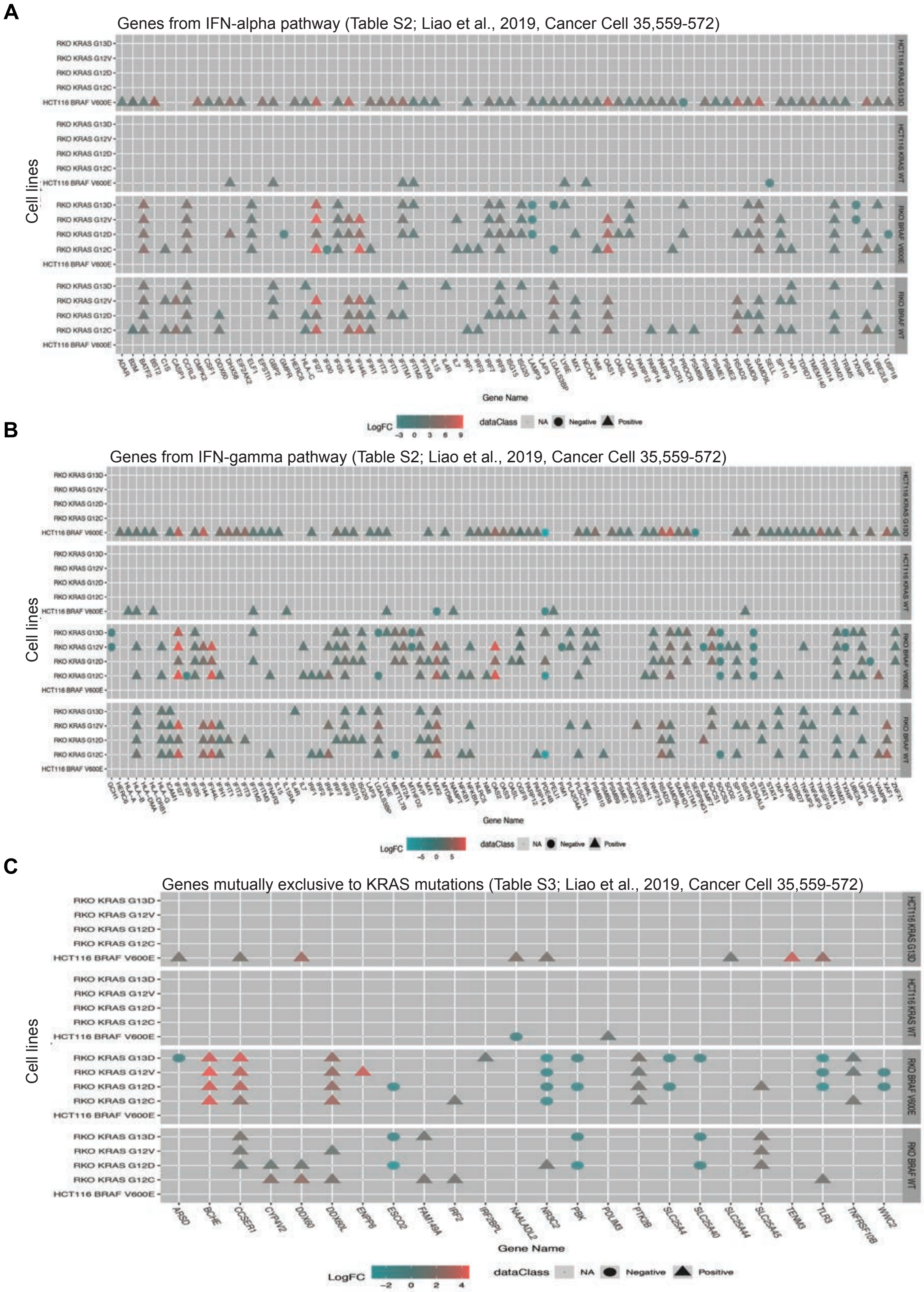

Supplementary figure 8. Differentially expressed immune related genes. DEGs with $|\log_2 \mathrm{FC}| > 1$ and adjusted $P < 0.05$ from transcriptome data were related to the IFN-alpha pathway (**A**) or IFN-gamma pathway (**B**) and immune genes whose genomic deletions were mutually exclusive to *KRAS* mutations (**C**).

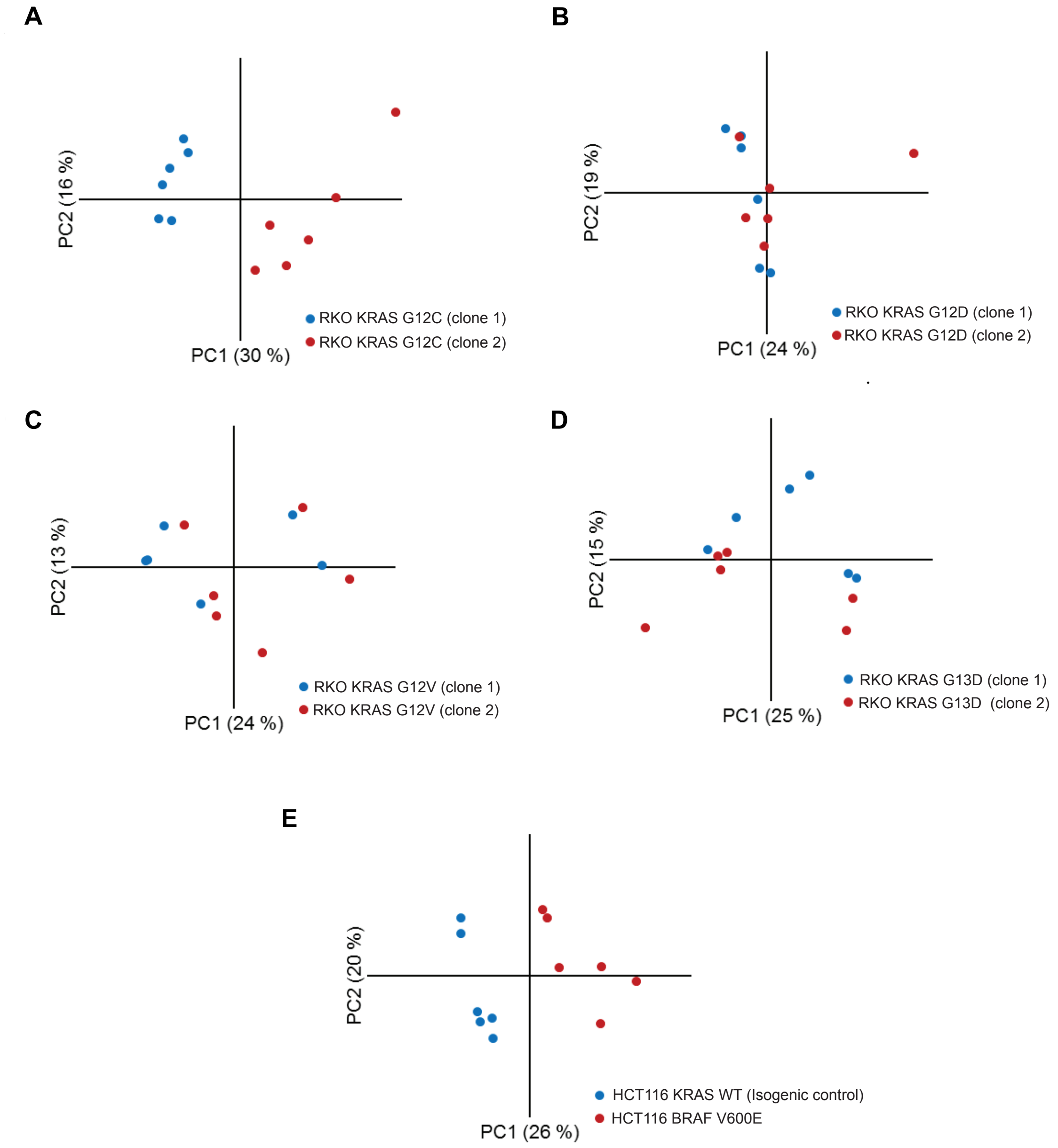

Supplementary figure 9. Similarity of cell clone metabolism between duplicate cell clones with the same *KRAS* knock-in mutation. Principal Component Analysis (PCA) of the metabolome datasets from the two included clones of KRAS G12C (**A**), G12D (**B**), G12V (**C**), G13D (**D**) and one clone each of (**E**) HCT116 KRAS WT and HCT116 BRAF V600E.

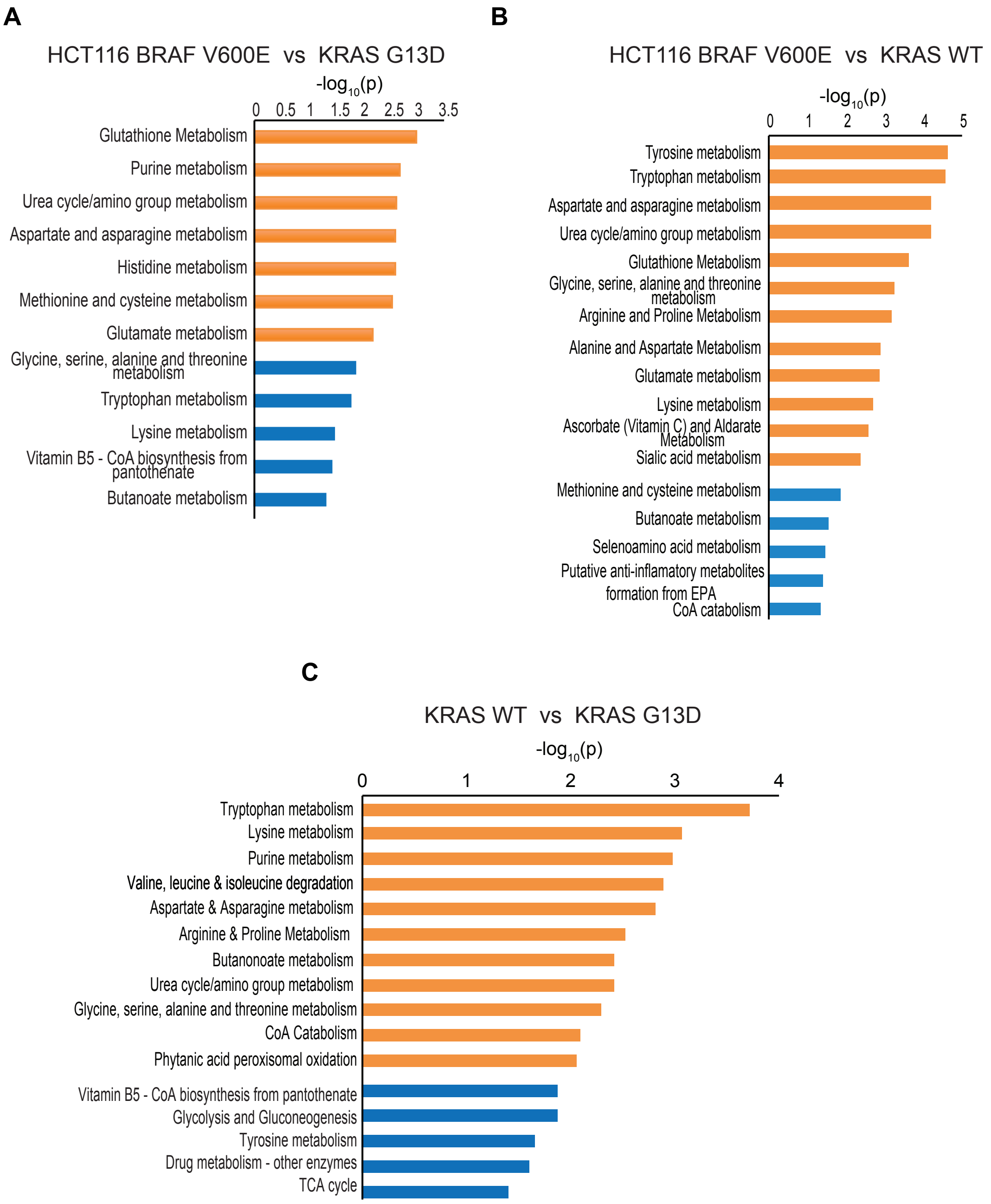

Supplementary figure 10. Amino acid metabolism is altered by *KRAS* and *BRAF* mutation in HCT116 cells. LC-MS based metabolomic analyses were performed using BRAF V600E knock-ins and isogenic control cells and subjected to pathway analysis comparing BRAF V600E vs KRAS G12D (**A**), BRAF V600E vs KRAS WT (**B**) and isogenic controls (**C**) cells. Pathways with $P < 0.001$ and 0.05 were designated with orange and blue bars, respectively.

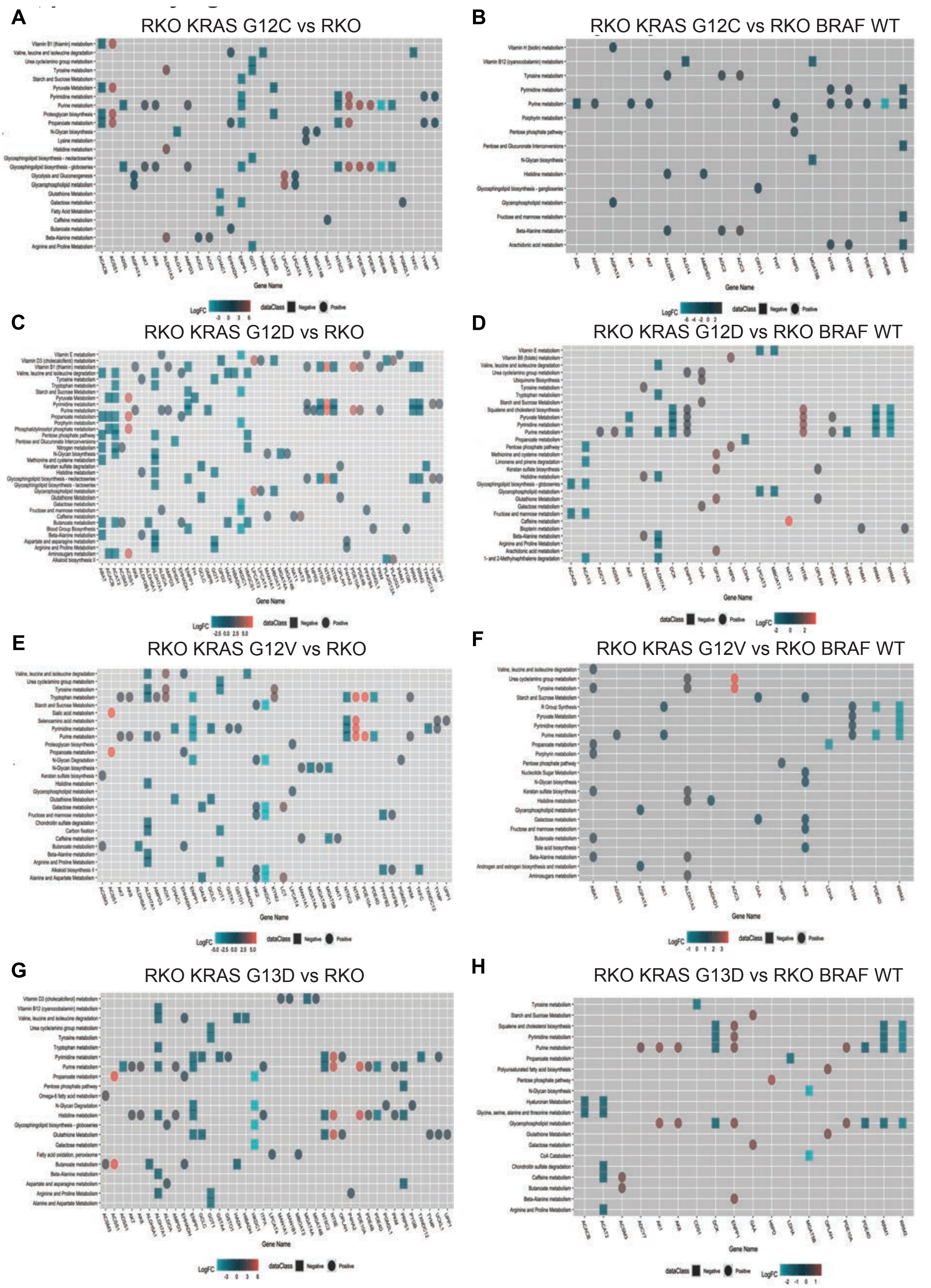
A
RKO KRAS G12C vs RKO
B
RKO KRAS G12C vs RKO BRAF WT
C
RKO KRAS G12D vs RKO
D
RKO KRAS G12D vs RKO BRAF WT
E
RKO KRAS G12V vs RKO
F
RKO KRAS G12V vs RKO BRAF WT
G
RKO KRAS G13D vs RKO
H
RKO KRAS G13D vs RKO BRAF WT
Gene Name
LogFC
dataClass
Negative
Positive

94

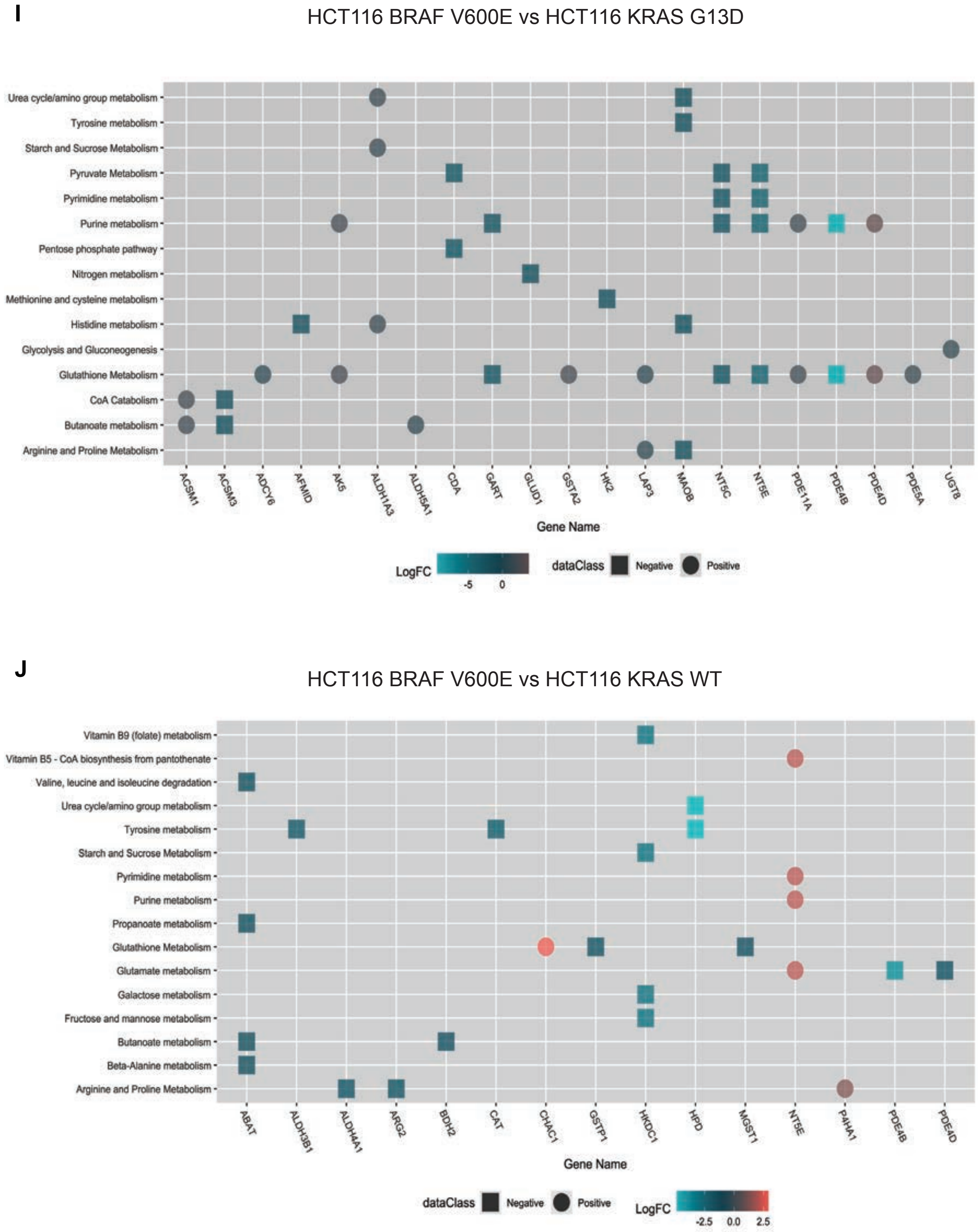

Supplementary figure 11. Metabolic pathway genes are deregulated in the transcriptomes of *KRAS* and *BRAF* V600E mutant cells. The enzymes for the substrate or product metabolites identified were searched in the KEGG database, and the genes encoding enzymes which belong to the respective pathway were extracted. The extracted genes were analyzed in the transcriptome data for their Differential Gene Expressions (DEGs) with log2 >1 and adjusted *P value < 0.05* from comparisons among RKO KRAS G12C/D/V/13D vs RKO BRAF V600E (**A, C, E, G**), RKO BRAF wild-type (**B, D, F, H**), HCT116 BRAF V600E vs HCT116 KRAS G13D (**I**) and HCT116 BRAF V600E vs HCT116 KRAS wild-type (**J**).

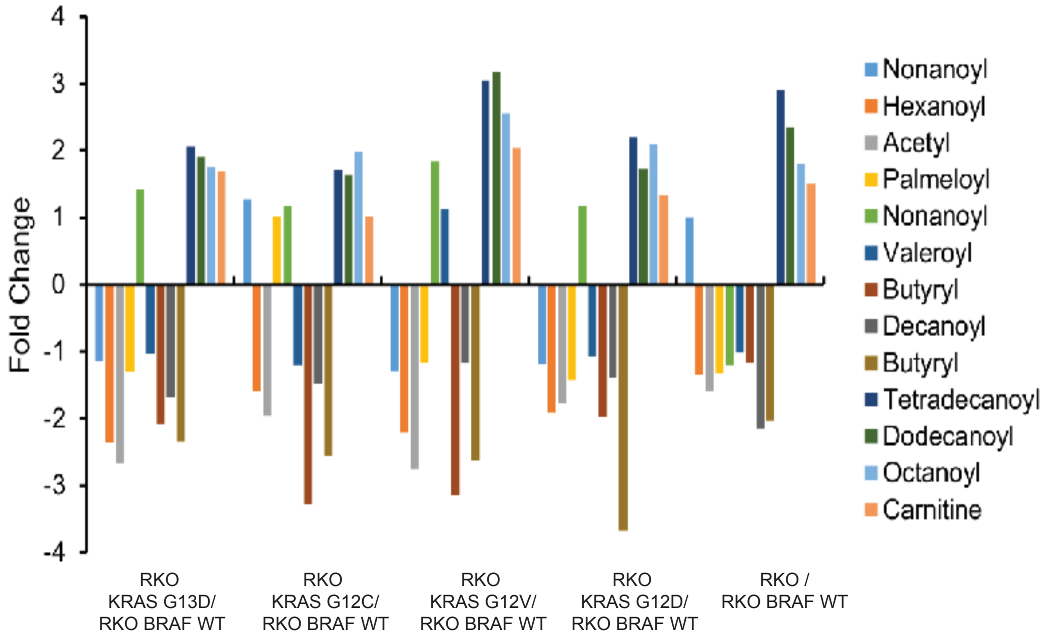

Supplementary figure 12. Carnitine levels in knock-ins of *KRAS* G12C/D/V/13D. The majority of all detected acylated carnitines were decreased in *KRAS* and *BRAF* mutant clones whereas tetradecanoyl, docanoyl and octanoyl carnitine were more abundant.

Additional File 2: Supplementary tables' legends

Supplementary table 1. Primers used for isogenic cell model generation and validation. List of primers for generating isogenic cell models for different KRAS and BRAF mutations using rAAV mediated genome editing (2A). Primers for validations by Sanger sequencing for presence of mutations of interest of KRAS and BRAF (2B) and the validation results (2C).

Supplementary table 2. Differentially Expressed Genes (DEGs) in *KRAS* and *BRAF* isogenic cell models. Differentially expressed genes (DEGs) with $|\log_2 FC| > 1$ and FDR ≤ 0.05 from comparisons of RKO cells with *KRAS* G12C/D/V/G13D to wildtype (2A, C, E, G) or *BRAF* V600E (2B, D, F, H) and HCT116 BRAF V600E to HCT116 (2I) or HCT116 KRAS WT (2J) . FDR, False Discovery Rate; $\log_2$FC, log2 transformed fold change of gene expression.

Supplementary table 3. Differentially Expressed Genes (DEGs) from overlapped Venn regions (Supplementary figure 5A-D and F) and their trend of expression in the transcriptome datasets with logFC values. Differentially expressed genes (DEGs) with $|\log_2 FC| > 1$ and FDR ≤ 0.05 from comparisons of RKO cells with *KRAS* G12C/D/V and G13D to wildtype or *BRAF* V600E were identified in the overlapped regions of the Venn diagramme analysis (Supplementary figure 5A-D and F).The "Trend of expressions" were determined as all negative, negative $\log_2$FC value in both of the comparisons; all positive, positive $\log_2$FC value in both of

the comparisons and mixed, negative and positive log_2FC value in one or another. Here, FDR, False Discovery Rate; log_2FC, log2 transformed fold change of gene expression.

Supplementary table 4. Expression status of Differentially Expressed Genes (DEGs) from *KRAS* and *BRAF* isogenic cell models in TCGA CRC samples harboring different *KRAS* mutations. The representations of DEGs from our transcriptome datasets into TCGA transcriptome datasets of colon and rectum samples harboring KRAS G12/C/D/V/13D mutation in comparisons to samples with no mutations in this study. Here, TCGA log_2FC, the log2FC from TCGA data; DiffExpr log_2FC, log_2FC from our data and Directionality, directionality of the expression (positive, if log_2FC values in both TCGA and our dataset are positive; negative, if log_2FC values in both TCGA and our dataset are negative and opposing, if log2FC values in both TCGA and our dataset are positive and negative or vice versa).

Supplementary table 5. Differentially Expressed Proteins (DEPs) in *KRAS* and *BRAF* isogenic cell models. Differentially expressed proteins with $|\log_2 FC| \geq 1$ and adjusted P value $\leq 0{,}05$ from comparisons of *KRAS* G12C/D/V and G13D to wildtype (3A, C, E, G) or *BRAF* V600E (3B, D, F, H) and HCT116 BRAF V600E to KRAS G13D. log_2FC, log_2-transformed fold change of the expression between contrast groups; AveExpr , Average protein expression; t, moderated t-statistics; P.Value, p-value; Adj.P.val , Benjamini-Hochberg adjusted p-value; B, log-odds of the protein being differentially expressed.

Supplementary table 6. Pathway analysis of differentially expressed genes (DEGs) in *KRAS* mutant RKO cells. Ingenuity Pathway Analysis (IPA) of differentially expressed genes with $|\log_2 FC| > 1$ and FDR ≤ 0.05 from *KRAS* G13D, G12C/D/V knock-ins compared to wild-type and *BRAF* V600E (5A-D). -log(p-value), $-log_{10}$ of the Benjamini-Hochberg corrected p-value obtained from the Fisher's Exact test; zScore, A score indicating predicted activation (z-score>2) or inactivation (z-score < -2) of a pathway in question ; Ratio , no. of differentially expressed genes in a given pathway divided by the total number of genes that make up that pathway and that are in the reference gene set; P-Value, reverse log of the $-log_{10}$ p-values; geneNames, HGNC symbols of the differentially expressed genes associated with the pathway in question; Adj. p-value , p-values adjusted for multiple testing using the Benjamini-Hochberg procedure and cut-off <0,05.

Supplementary table 7. Pathway analysis of differentially expressed proteins (DEPs) in KRAS mutant RKO cells. Ingenuity Pathway Analysis (IPA) of differentially expressed genes with $|\log_2 FC| > 1$ and FDR ≤ 0.05 from comparisons of KRAS G12D and G13D to wild-type and BRAF V600E cells (6A-B). -log(p-value), $-log_{10}$ of the Benjamini-Hochberg corrected p-value obtained from the Fisher's Exact test; zScore, A score indicating predicted activation (z-score>2) or inactivation (z-score < -2) of a pathway in question ; Ratio , no. of differentially expressed genes in a given pathway divided by the total number of genes that make up that pathway and that are in the reference gene set; P-Value, reverse log of the $-log_{10}$ p-values; geneNames, HGNC symbols of the differentially expressed genes associated with the pathway in question; Adj. p-value, p-values adjusted for multiple testing using the Benjamini-Hochberg procedure and cut-off <0,05.

Supplementary table 8. Immune related DEGs in KRAS mutant RKO cells. The status of DEGs related to IFN-alpha pathway (Supplementary table 2; Liao et al., 2019) (8A), IFN-Gamma pathway (Supplementary table 2; Liao et al., 2019) (8B) and immune genes whose genomic deletions were mutually exclusive to KRAS mutations

(Supplementary table 3; Liao et al., 2019) (8C) in our transcriptome dataset. DiffExpr Log_2FC, $-log_2FC$ from our transcriptome data.

Supplementary table 9. Enrichment of metabolic pathways in KRAS mutant RKO cells. LC-MS metabolomics data were acquired in both positive and negative mode from KRAS G12C/D/V/G13D knock-ins compared to BRAF V600E or wild-type (9A-H) and HCT116 BRAF V600E to KRAS G13D as well as KRAS WT (9I-J).The Mummichog python package was used to perform pathway analysis on each dataset and the positive mode and negative mode results were combined using Fisher's method to produce a final p-value (a p-value threshold of 0.05 was applied).

Supplementary table 10. Enrichment of Differentially expressed Genes (DEGs) for altered metabolic pathways in *KRAS* and *BRAF* isogenic cell models. Differentially Expressed Genes (DEGs) for significantly altered metabolic pathways extracted from KEGG pathway database were analyzed on transcriptome dataset (Supplementary table 2A-J) for KRAS G12C/D/V/G13D knock-ins compared to BRAF V600E or wild-type (10A-H) and HCT116 BRAF V600E to KRAS G13D as well as KRAS WT (10I-J) with log2 fold change ≥ 1 and FDR ≤ 0,05. FDR, False Discovery Rate; logFC, log_2-transformed fold change of expression.
